# Supplementary figures and images for: Study of the thermal regime of a reservoir on the Qinghai-Tibetan Plateau, China
Source: PLoS One. 2020 Dec 21;15(12):e0243198. doi: 10.1371/journal.pone.0243198 (PMC7751983; doi:10.1371/journal.pone.0243198)

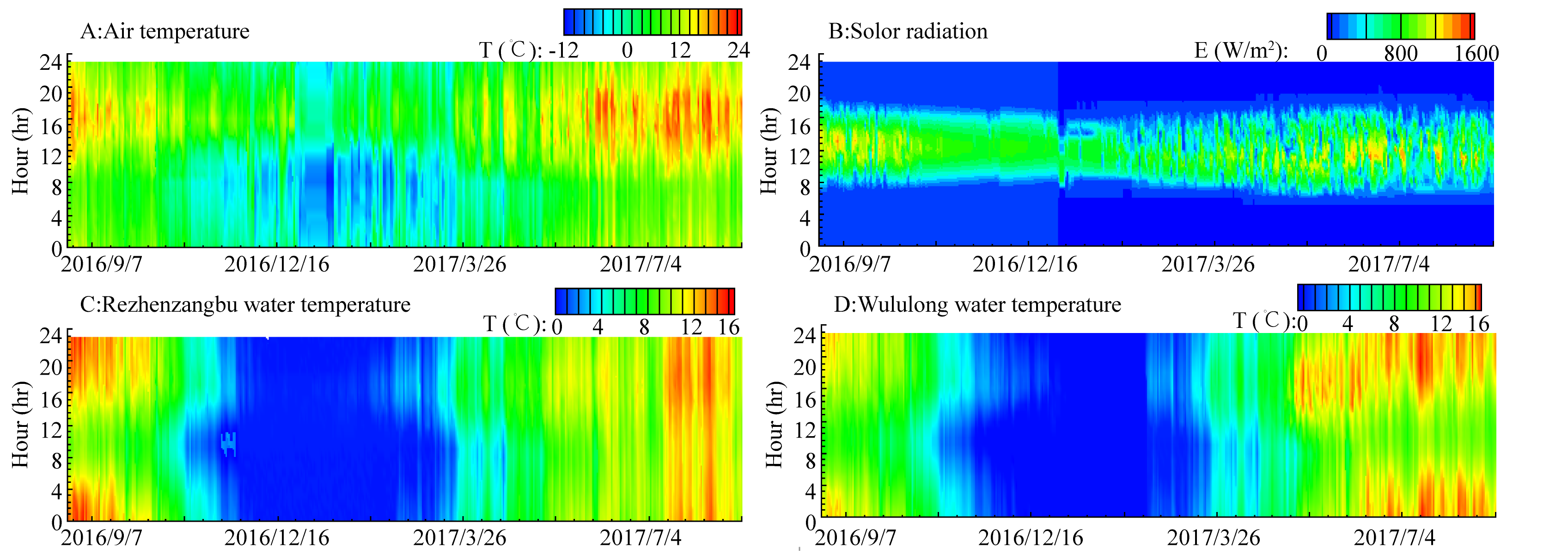

Supplement: S1 Fig — Temporal distribution of measured air temperature (A), solar radiation (B), and inflow water temperature (C Rezhenzangbu and D Wululong) in the study area. (TIF) [file pone.0243198.s001.tif]

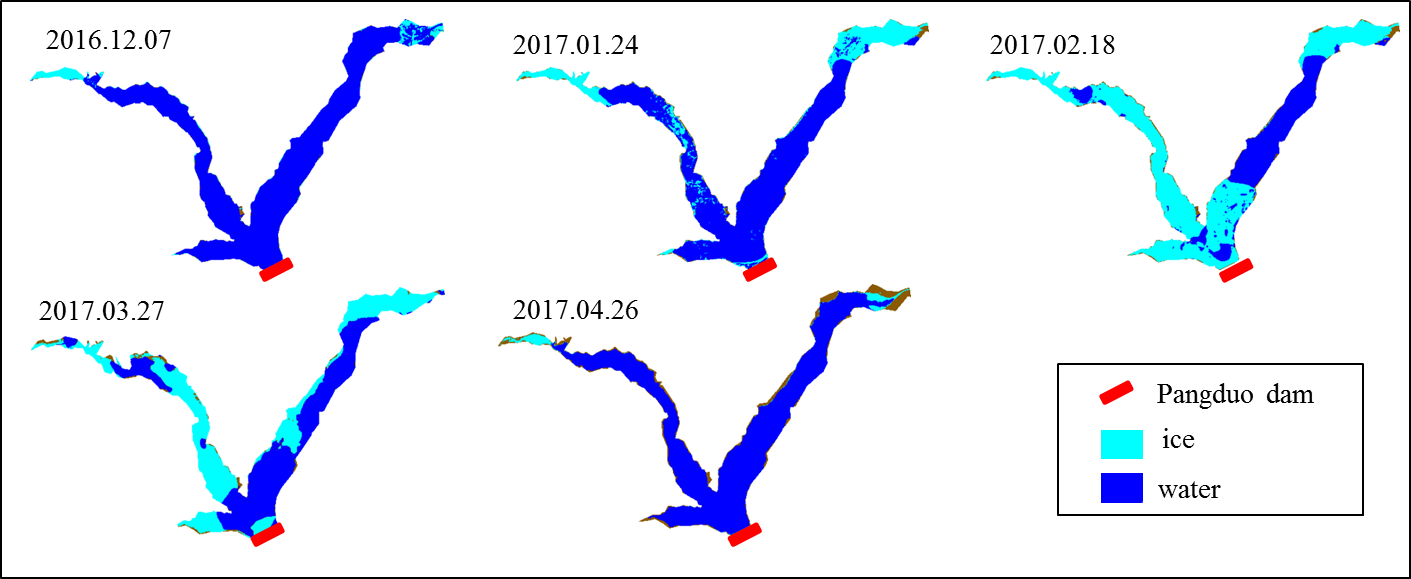

Supplement: S2 Fig — (TIF) [file pone.0243198.s002.tif]

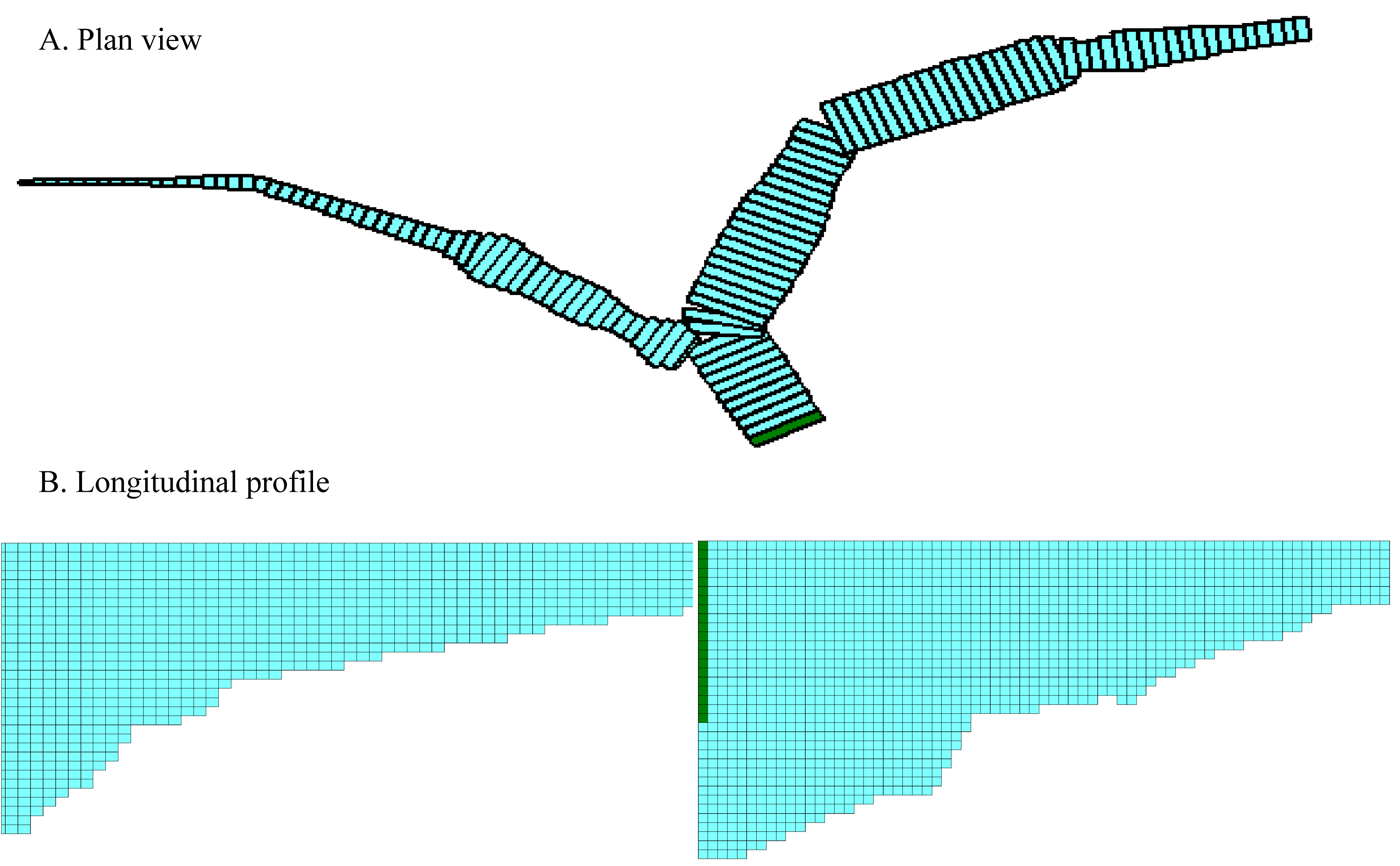

Supplement: S3 Fig — (TIF) [file pone.0243198.s003.tif]

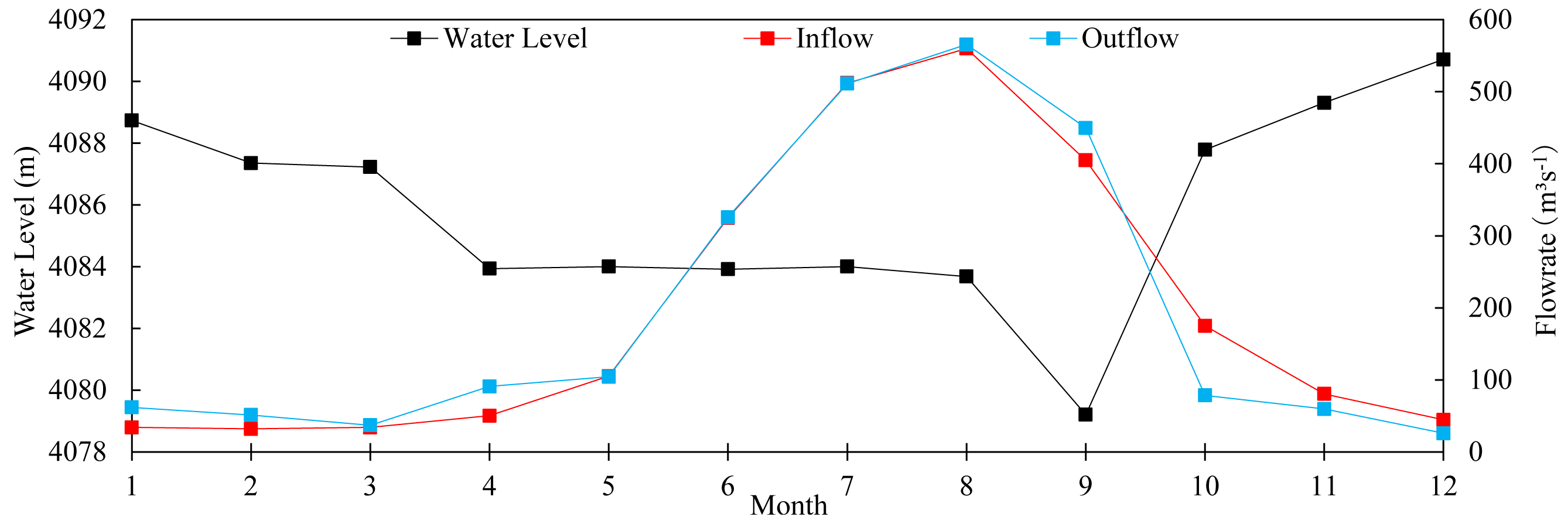

Supplement: S4 Fig — (TIF) [file pone.0243198.s004.tif]

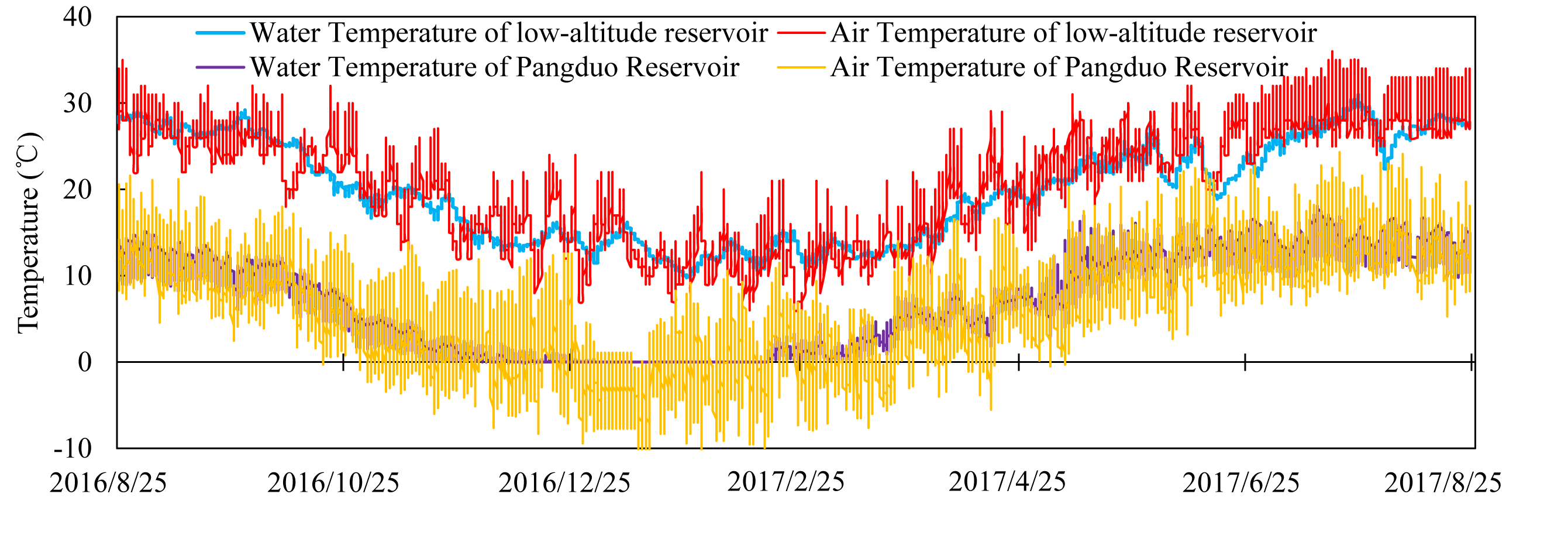

Supplement: S5 Fig — (TIF) [file pone.0243198.s005.tif]

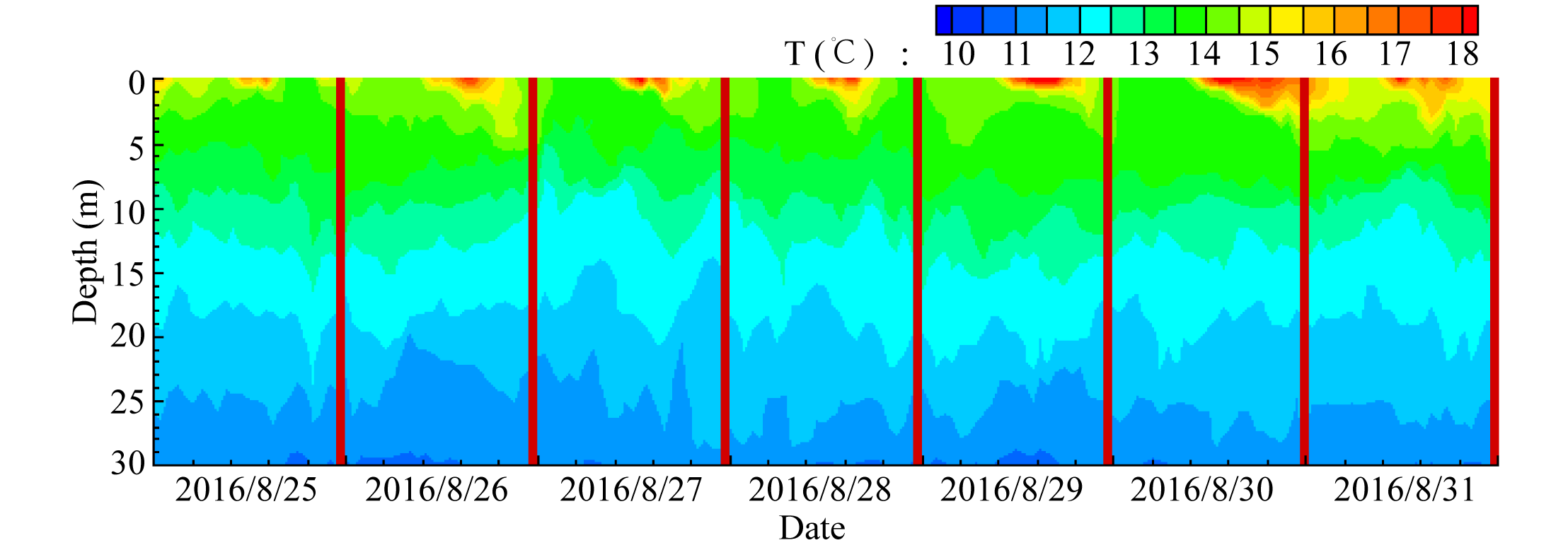

Supplement: S6 Fig — (TIF) [file pone.0243198.s006.tif]

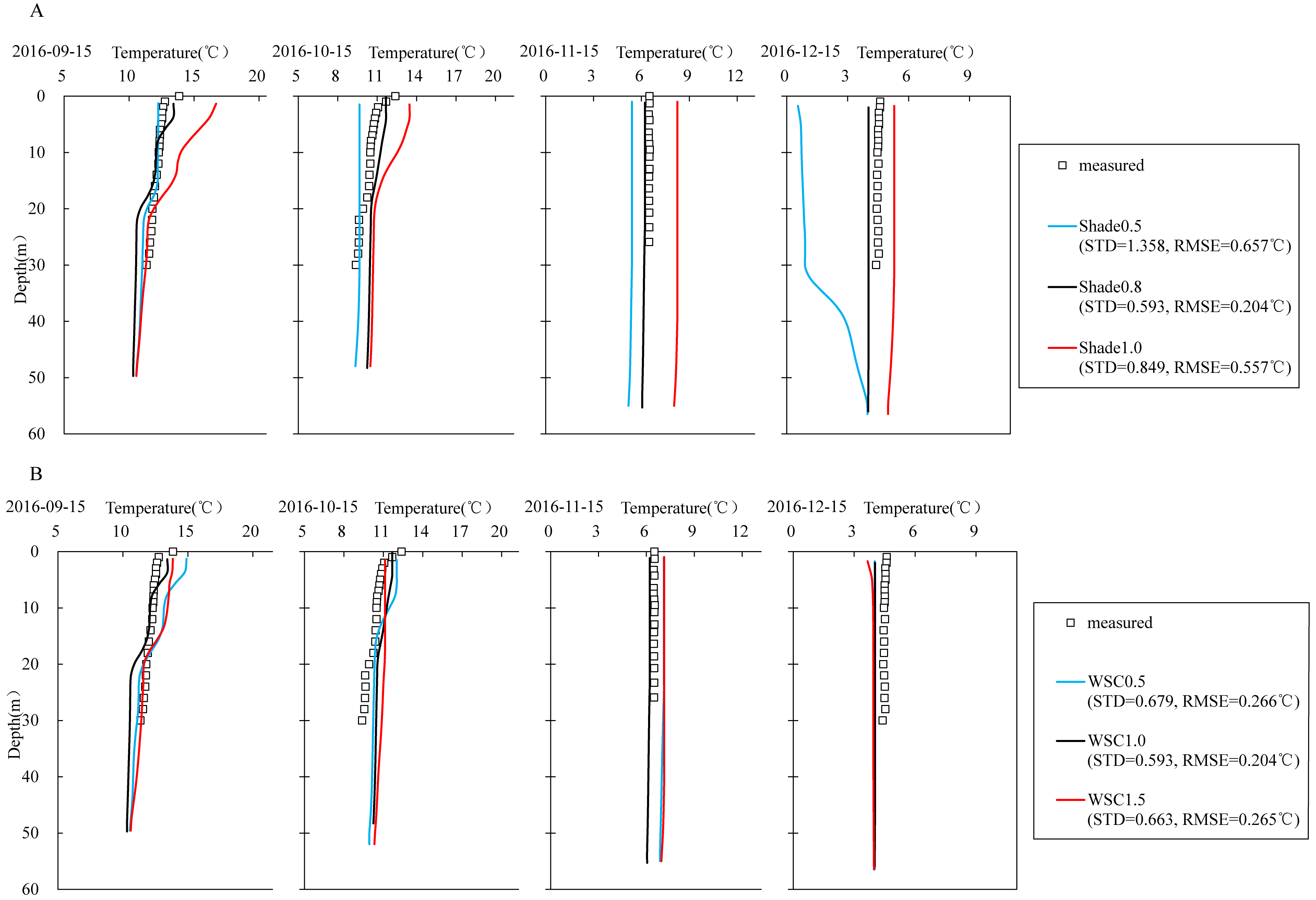

Supplement: S7 Fig — Simulated temperature profiles at the section upstream to the Pangduo dam using three shades (A), three WSCs (B) and measured profiles on Sep. 15, 2016; Oct. 15, 2016; Nov. 22, 2016 and Dec. 15, 2016. (TIF) [file pone.0243198.s007.tif]

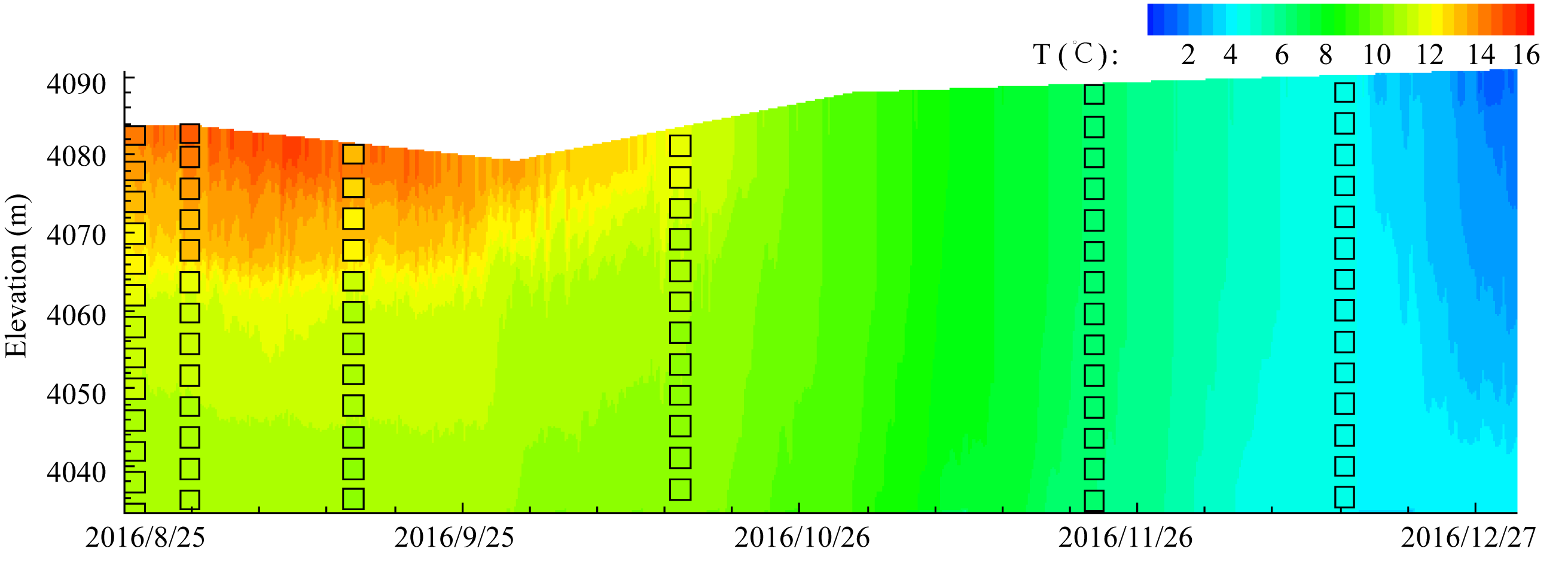

Supplement: S8 Fig — (TIF) [file pone.0243198.s008.tif]

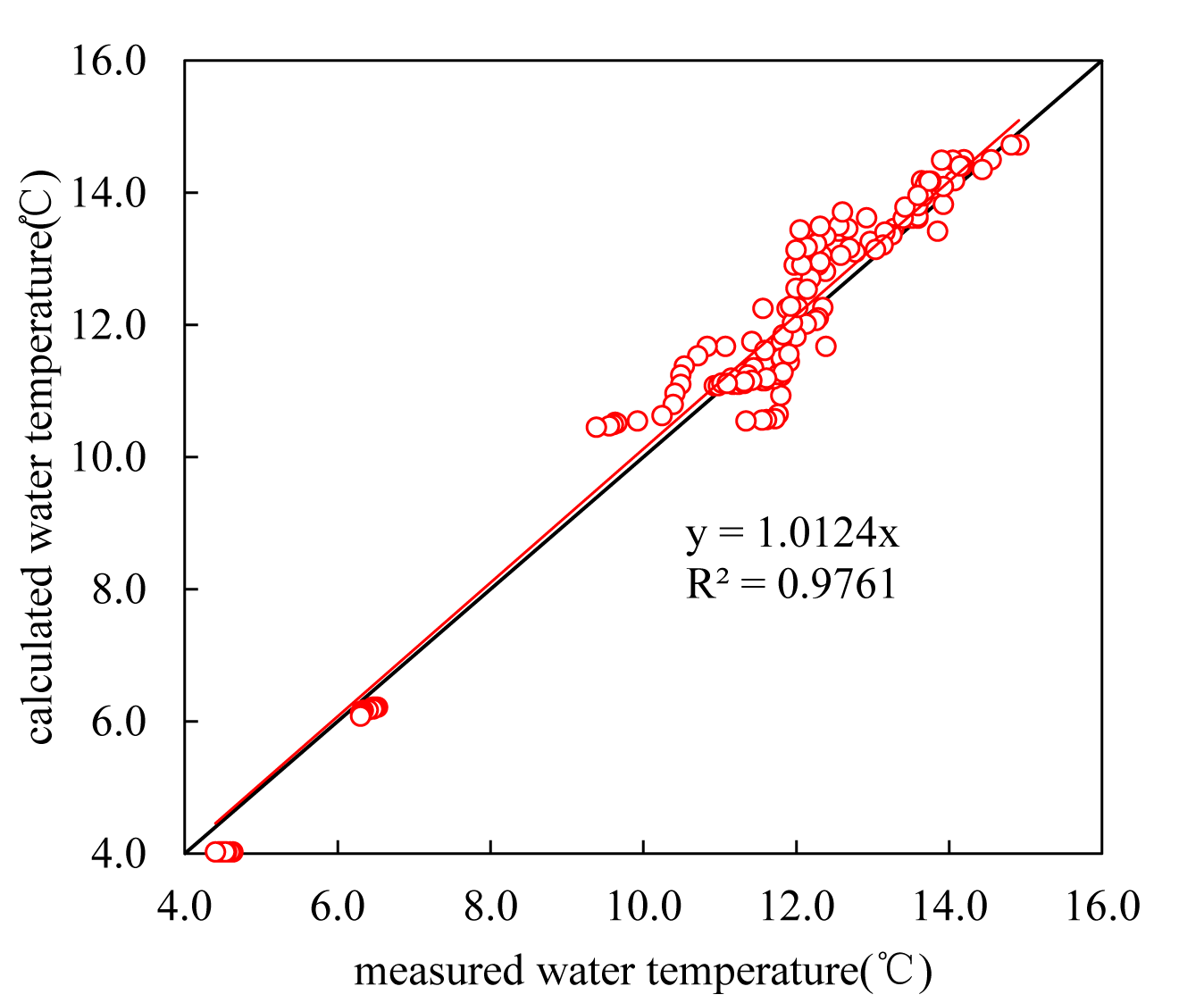

Supplement: S9 Fig — (TIF) [file pone.0243198.s009.tif]

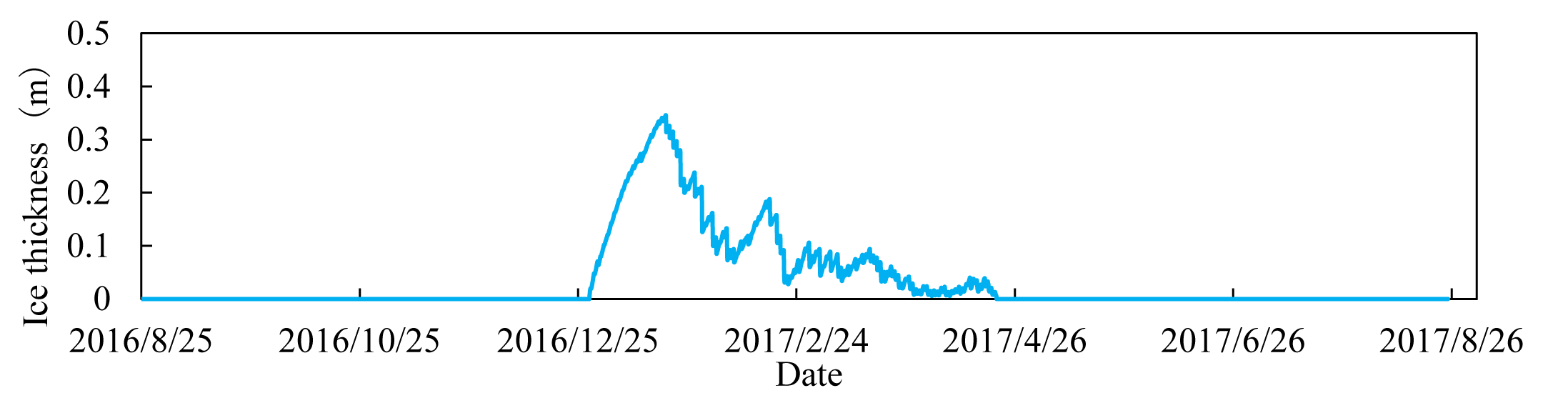

Supplement: S10 Fig — (TIF) [file pone.0243198.s010.tif]

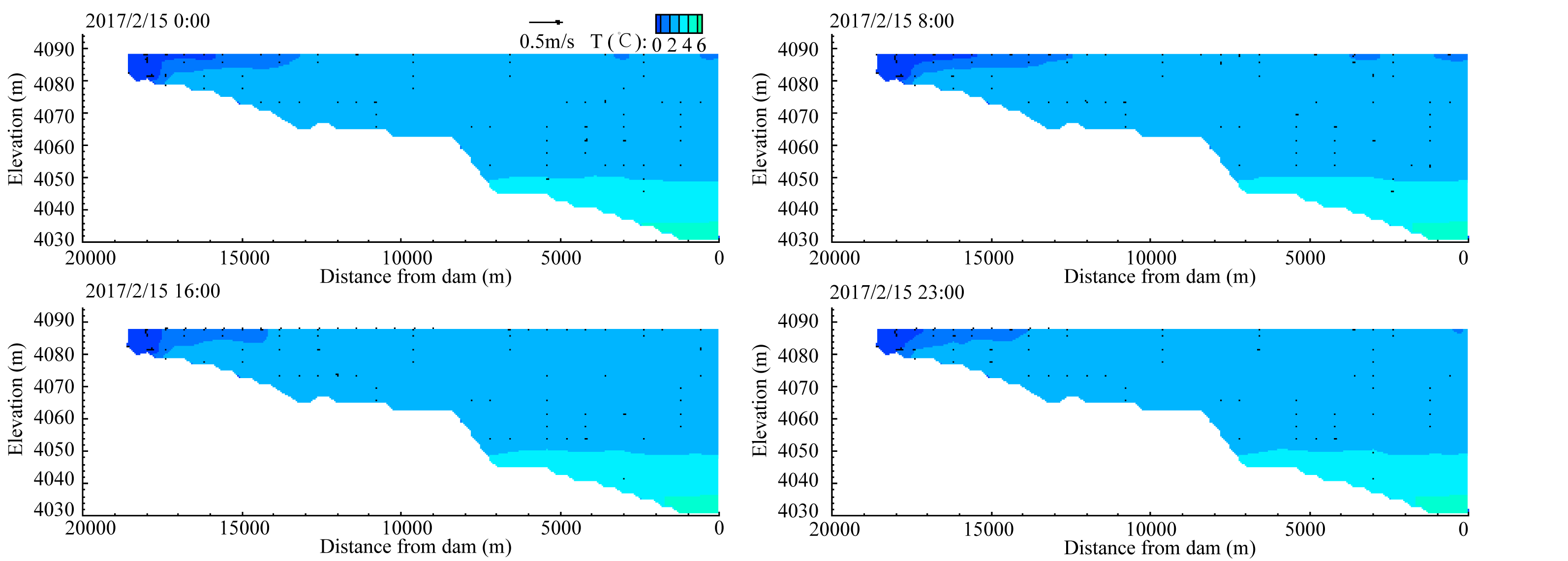

Supplement: S11 Fig — (TIF) [file pone.0243198.s011.tif]

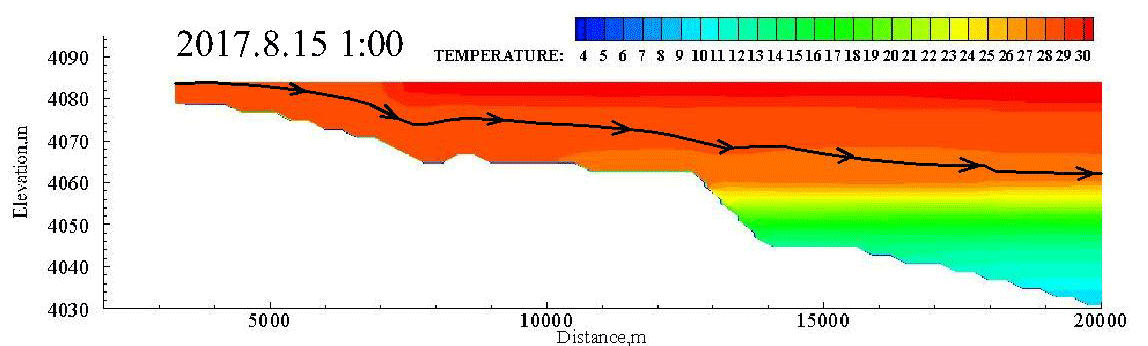

Supplement: S1 File — (ZIP) [file pone.0243198.s012.zip › Inflow mixing mode between the Pangduo Reservoir and the low-altitude reservoir/Low-altitude Reservoir.gif]

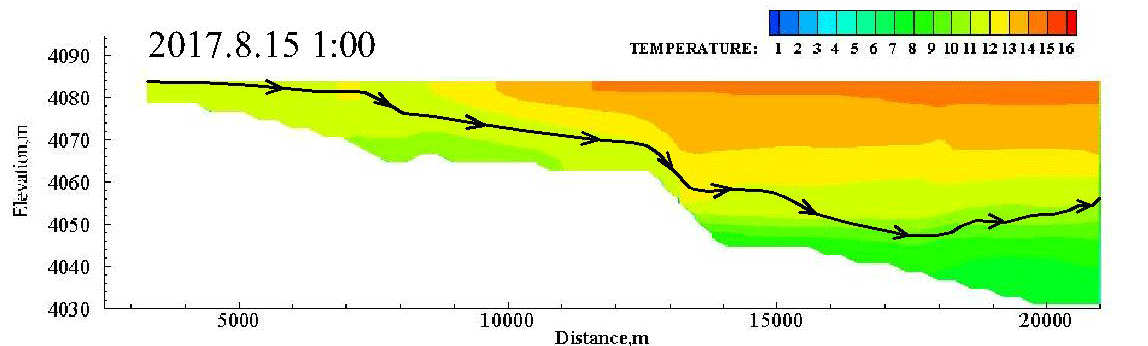

Supplement: S1 File — (ZIP) [file pone.0243198.s012.zip › Inflow mixing mode between the Pangduo Reservoir and the low-altitude reservoir/Pangduo Reservoir.gif]
