# Supplementary material for: Study of the thermal regime of a reservoir on the Qinghai-Tibetan Plateau, China
Source: PLoS One. 2020 Dec 21;15(12):e0243198. doi: 10.1371/journal.pone.0243198 (PMC7751983; doi:10.1371/journal.pone.0243198)
Supplement: Attachment — Submitted filename: Yang_et_al_2020.pdf [file pone.0243198.s014.pdf]

Response to: Study of the thermal regime of a reservoir on the Qinghai-Tibetan Plateau, China

General comments:

This study adapted CE-QUAL-w2 and simulated the 2-D sub-daily thermal structure of a high-altitude reservoir in Qinghai-Tibetan Plateau. The authors aim to further our understanding of a reservoir's diurnal variation of a dimictic reservoir. Generally speaking, this draft is very raw and requires much work. I will reject it. However, the topic is interesting so I recommend resubmission.

1. Please add section numbers.
2. The introduction requires much effort. Key issues are as follows:
    a. Literature review is not solid. It read like a stack of facts about reservoir temperatures. It is not clear what has been done and where the research gap is.
    b. The novelty of this study is not emphasized enough. Many studies have been using CE-QUAL-w2 to study the thermal structure of a reservoir. Is the novelty of this study to apply CE-QUAL-w2 in a reservoir in Qinghai-Tibetan Plateau? What is so special about this reservoir?
    c. Assertive statements without reference. See specific comments below.
3. The choice of three metrics, i.e., *VTG*, *N*, and *SI*, is redundant and not well justified.
4. Language needs to be polished. A few things came up frequently
    a. Be very careful with the word "significant". If you did not conduct a statistical significance test, don't use it [Line 18, 198, 280, 329…].
    b. Please avoid using "obvious".
    c. Please avoid using descriptive terms, e.g., this model "accurately" captures… [Line 15, 218]. Please simply provide the summary of model performance and readers can judge whether the model performance is satisfactory or not.
5. Poor interpretation of model results. The authors exhaustively presented model results while those results were not interpreted carefully. For example, how to properly understand *SI*? Does larger *SI* value denote more stable or less stable water column? How is *SI* different than buoyance frequency?

Specific comments:

Abstract

Line 9: "*...meteorological characteristics with low temperature...*" it is better to clarify 'low air temperature' because it can be confused with reservoir water temperature.

Line 13: Please simply explain what a "dimictic reservoir" is. This term is not commonly acknowledged.

Line 14-15: Please provide a summary of your model performance. Do not use the statement, i.e., "the model **accurately** simulated…". Readers can determine whether your model is accurate or not based on your model performance summary.

Introduction

Line 54: “Moreover, early research focused primarily on regional differences.” I don’t think this is correct.

Line 56: “however, the lack of detailed data samples and perfect research methods reflect certain existing limitations” This statement is ambiguous, and no research methods are perfect.

Line 62-64: Wordy. I would recommend changing to “the Qinghai-Tibetan Plateau is ecologically vulnerable to human activities and environmental changes.”

Line 69: “few scientists **seem to** be aware”. It is not proper to use “seem to be” in a scientific paper.

Some sentences are very long and hard to follow, e.g., Line 47-49, Line 72-75, and Line 76-79.

Study area:

Line 85: What is a “controlling project”?

Line 86: What is a normal storage level? Is it necessary in this study?

Line 87-88: Unit for “total water area” is wrong. Additionally, the water area and depth in front of dam should vary with the amount of water stored in reservoirs.

Line 97: Please switch Rezhenzangbu and Wululong to be consistent with Figure numbering.

Table 1: B1: Please use “Upstream of dam” instead of “In front of dam”.

Mathematical models and methods

Line 119: “~~clear characteristics of~~ strong diurnal variation”

Line 119-121: Try not to use passive voice.

Line 123: What variables are you referring to?

Line 133: “…, $B\eta$ the water surface width” where η should be subscript

Line 134: is S_{Φ} and φ_{GY} the same thing? If so, can you make the notation consistent in this paper?

Line139: please add reference for this equation.

Line 144: Following terms i.e., the longitudinal eddy viscosity coefficient and the longitudinal eddy current diffusion coefficient, do not occur anywhere in previous context. Are you referring to longitudinal and vertical dispersion coefficients as in Line 134? If so, please be consistent with terminology throughout the paper. Additionally, please explain why you chose those two values.

Line 146: What is formula W2N?

Line 164: "Although N is the convention used in limnology and oceanography, reservoirs are similar." The buoyancy frequency has been used in manmade reservoirs for a very long time, e.g., Snodgrass& O'Melia (1975), Niemeyer et al., (2018).

Eqn 11: I think it is more generalizable to use an integral instead of summing.

Line 177-179: I don't understand.

Results and discussion

Line 182: Mean annual air temperature? Be specific

Line 186-189: You mentioned solar radiation twice with description of longwave radiation in between. Please do not go back and forth and explain solar radiation first and then longwave radiation. Also why do you only explain the diurnal variation of solar radiation only? Why not talk about the diurnal variation of longwave radiation?

Line 193: Define "daily variation". How do you calculate that? This is an important variable and used many times in this study. A definition is necessary.

Line 198: Be careful with the word "significant". This word usually implies that the authors have conducted a statistical significance test and the trend/result they find is statistically "significant". "Strong diurnal variation" is better.

Line 210-212 As shown in Table 1, measured vertical water temperature was available from 08/25/2016 to 08/31/2016. How did you get measured temperature data for 09/15, 10/15, 11/22, and 12/15? Additionally, as shown in Figure 3, the temperature difference between depth at 0 m and depth at 30 m can be as large as 8 degree Celsius. It is incorrect to say, "temperature stratification of the reservoir is weak," since reservoirs should be more stratified at warmer seasons. Based on Section **Monitoring data analysis**, hottest month is July, so it would make the model more convincing if we can see the model performance for a hot July day. I am not sure whether that data is available or not, but an alternative way is to show the model performance for 08/25 to 08/31 when thermal stratification is stronger than 09/15.

Line 215-216: The metric shown in the paper is also misleading since the errors can be diluted by the winter days when thermal stratification is weak. For example, we can see that, on 09/15/2016, model simulated a stronger stratification than the measurement(Figure 4).

Figure 5: I cannot tell the model performance from this plot. This only shows the model simulation result. Please show measurement in this plot.

Line 240: Be consistent with the unit of Buoyance Frequency (*N*). You defined the unit of *N* as 1/s in Line 159.

Line 249-250: Expression is unprofessional. Please rephrase.

Line 269: Sooner than what?

Line 269-270: The retention time of reservoirs is irrelevant to elevations. Retention time is a measure of how long water resides in reservoirs, roughly depending on reservoir storage and outflow. Reservoirs at low altitude can have either very short or very long residence times. You may refer to Cheng et al. (2020), Yearsley et al. (2019), Yigzaw et al. (2019)

Line 280: If the authors did not conduct statistical significance test, please use “strong” or other words instead of “significant”.

Line 285: If the time step is hourly, the minutes are not necessary, i.e., 8AM will do. Additionally, please use 12AM instead of 0AM.

Second paragraph in Section **Diurnal characteristics analysis** is poorly organized and hard to follow. The authors jump back-and-forth between top 5-m range and top 20-m range, so the values are hard to be compared across different time slots.

Figure 7 is boring and not informative.

Line 310-311: Recommend not using one sentence as a paragraph.

Figure 9 is boring. The authors can simply say that diurnal variation is weak in winter.

Figure 10: Why is there no shortwave radiation from 12/23/2016 to 04/22/2017? Figure S1 shows that at least one-third of reservoir surface was not covered with ice even at coldest time (02/18/2017). In CE-QUAL-w2 model, did you assume that all reservoir surfaces are uniformly covered with ice?

Line 356: How to interpret *SI* value? Does bigger value mean less stability?

Reference:

Cheng, Y., Voisin, N., Yearsley, J. R., & Nijssen, B. (2020). Reservoirs modify river thermal regime sensitivity to climate change: a case study in the southeastern United States. Water Resources Research, 56(6), e2019WR025784.

Niemeyer, R. J., Cheng, Y., Mao, Y., Yearsley, J. R., & Nijssen, B. (2018). A thermally stratified reservoir module for large-scale distributed stream temperature models with application in the Tennessee River Basin. Water Resources Research, 54(10), 8103-8119.

Snodgrass, W. J., & O’Melia, C. R. (1975). Predictive model for phosphorus in lakes. Environmental Science & Technology, 9(10), 937–944. https://doi.org/10.1021/es60108a005

Yearsley, J., Sun, N., Baptiste, M., & Nijssen, B. (2019). Assessing the impacts of hydrologic and land use alterations on water temperature inthe Farmington River basin in Connecticut. Hydrology and Earth System Sciences, 23(11), 4491–4508. https://doi.org/10.5194/hess-23-4491-2019

Yigzaw, W., Li, H. Y., Fang, X., Leung, L. R., Voisin, N., Hejazi, M. I., & Demissie, Y. (2019). A Multilayer Reservoir Thermal Stratification Module for Earth System Models. Journal of Advances in Modeling Earth Systems, 11(10), 3265-3283.
