# Supplementary material for: Study of the thermal regime of a reservoir on the Qinghai-Tibetan Plateau, China
Source: PLoS One. 2020 Dec 21;15(12):e0243198. doi: 10.1371/journal.pone.0243198 (PMC7751983; doi:10.1371/journal.pone.0243198)
Supplement: Attachment — Submitted filename: PONE-D-20-19898_reviewer.pdf [file pone.0243198.s015.pdf]

PLOS ONE

Study of the thermal regime of a reservoir on the Qinghai-Tibet Plateau, China

--Manuscript Draft--

**Manuscript Number:**	PONE-D-20-19898
**Article Type:**	Research Article
**Full Title:**	Study of the thermal regime of a reservoir on the Qinghai-Tibet Plateau, China
**Short Title:**	The thermal regime of a reservoir on the Qinghai-Tibet Plateau
**Corresponding Author:**	Yun Deng<br>Sichuan University<br>CHENGDU, SICHUAN CHINA
**Keywords:**	Qinghai-Tibetan Plateau; Reservoir thermal regime; Numerical modeling; Temperature diurnal variation
**Abstract:**	The Qinghai-Tibetan Plateau region has unique meteorological characteristics with low temperature, low air pressure, low humidity, little precipitation, and strong diurnal variation. The features of reservoir stratification change with thermodynamic processes under different climate and hydrological conditions. To explore the typical thermal regime of stratified reservoirs on the Qinghai-Tibetan Plateau, we simulated water temperature structures in Pangduo Reservoir, which is a typical dimictic reservoir on the Qinghai-Tibetan Plateau, adopting a two-dimensional numerical model. Field data was used to verify the model, and results showed that the model accurately simulated the vertical water temperature structure through a year as well as the whole ice period during winter. During the heating and the high-temperature period among a year, thermocline depth showed an evident diurnal variation within 20 m below the surface, inducing the deepening of surface mixing layer in the reservoir. However, the characteristics of diurnal variation were not significant in winter due to the influence of ice cover. Compared to reservoirs in low-altitude areas at the same latitude, Pangduo Reservoir behaved a shorter stratification period and weaker stratification stability, and the unique shortwave radiation process was majorly responsible for that according to the heat budget analysis. This study provided a scientific understanding of the thermal changes in stratified reservoirs under the special geographical and meteorological conditions on the Qinghai-Tibetan Plateau. Moreover, this model can serve as a reference for decisions related to the adaptive management of similar reservoirs on high-altitude areas.
**Order of Authors:**	Yanjing Yang
	Yun Deng
	Youcai Tuo
	Jia Li
	Tianfu He
	Min Chen
**Additional Information:**	
**Question**	**Response**
**Financial Disclosure**<br><br>Enter a financial disclosure statement that describes the sources of funding for the work included in this submission. Review the submission guidelines for detailed requirements. View published research articles from *PLOS ONE* for specific examples.	This work was supported by the National Key R&D Program of China (grant number2016YFC0502202) and the National Natural Science Foundation of China (grant numbers 91547211).

Powered by Editorial Manager® and ProduXion Manager® from Aries Systems Corporation

This statement is required for submission and **will appear in the published article** if the submission is accepted. Please make sure it is accurate.<br><br>**Unfunded studies**<br>Enter: *The author(s) received no specific funding for this work.*<br><br>**Funded studies**<br>Enter a statement with the following details:<br>• Initials of the authors who received each award<br>• Grant numbers awarded to each author<br>• The full name of each funder<br>• URL of each funder website<br>• Did the sponsors or funders play any role in the study design, data collection and analysis, decision to publish, or preparation of the manuscript?<br>• **NO** - Include this sentence at the end of your statement: *The funders had no role in study design, data collection and analysis, decision to publish, or preparation of the manuscript.*<br>• **YES** - Specify the role(s) played.<br><br>* typeset	
**Competing Interests**<br><br>Use the instructions below to enter a competing interest statement for this submission. On behalf of all authors, disclose any competing interests that could be perceived to bias this work—acknowledging all financial support and any other relevant financial or non-financial competing interests.<br><br>This statement **will appear in the published article** if the submission is accepted. Please make sure it is accurate. View published research articles from *PLOS ONE* for specific examples.	The authors have declared that no competing interests exist.

Powered by Editorial Manager® and ProduXion Manager® from Aries Systems Corporation

**NO authors have competing interests**<br><br>Enter: *The authors have declared that no competing interests exist.*<br><br>**Authors with competing interests**<br><br>Enter competing interest details beginning with this statement:<br><br>*I have read the journal's policy and the authors of this manuscript have the following competing interests: [insert competing interests here]*<br><br>* typeset	
**Ethics Statement**<br><br>Enter an ethics statement for this submission. This statement is required if the study involved:<br><br>• Human participants<br>• Human specimens or tissue<br>• Vertebrate animals or cephalopods<br>• Vertebrate embryos or tissues<br>• Field research<br><br>Write "N/A" if the submission does not require an ethics statement.<br><br>General guidance is provided below. Consult the submission guidelines for detailed instructions. **Make sure that all information entered here is included in the Methods section of the manuscript.**	N/A

Powered by Editorial Manager® and ProduXion Manager® from Aries Systems Corporation

**Format for specific study types**<br><br>**Human Subject Research (involving human participants and/or tissue)**<br>• Give the name of the institutional review board or ethics committee that approved the study<br>• Include the approval number and/or a statement indicating approval of this research<br>• Indicate the form of consent obtained (written/oral) or the reason that consent was not obtained (e.g. the data were analyzed anonymously)<br><br>**Animal Research (involving vertebrate animals, embryos or tissues)**<br>• Provide the name of the Institutional Animal Care and Use Committee (IACUC) or other relevant ethics board that reviewed the study protocol, and indicate whether they approved this research or granted a formal waiver of ethical approval<br>• Include an approval number if one was obtained<br>• If the study involved *non-human primates*, add *additional details* about animal welfare and steps taken to ameliorate suffering<br>• If anesthesia, euthanasia, or any kind of animal sacrifice is part of the study, include briefly which substances and/or methods were applied<br><br>**Field Research**<br><br>Include the following details if this study involves the collection of plant, animal, or other materials from a natural setting:<br>• Field permit number<br>• Name of the institution or relevant body that granted permission	
**Data Availability**<br><br>Authors are required to make all data underlying the findings described fully available, without restriction, and from the time of publication. PLOS allows rare exceptions to address legal and ethical concerns. See the PLOS Data Policy and FAQ for detailed information.	Yes - all data are fully available without restriction

A Data Availability Statement describing where the data can be found is required at submission. Your answers to this question constitute the Data Availability Statement and **will be published in the article**, if accepted.<br><br>**Important:** Stating 'data available on request from the author' is not sufficient. If your data are only available upon request, select 'No' for the first question and explain your exceptional situation in the text box.<br><br>Do the authors confirm that all data underlying the findings described in their manuscript are fully available without restriction?	
**Describe where the data may be found in full sentences. If you are copying our sample text, replace any instances of XXX with the appropriate details.**<br><br>• If the data are **held or will be held in a public repository**, include URLs, accession numbers or DOIs. If this information will only be available after acceptance, indicate this by ticking the box below. For example: *All XXX files are available from the XXX database (accession number(s) XXX, XXX.).*<br>• If the data are all contained **within the manuscript and/or Supporting Information files**, enter the following: *All relevant data are within the manuscript and its Supporting Information files.*<br>• If neither of these applies but you are able to provide **details of access elsewhere**, with or without limitations, please do so. For example:<br><br>*Data cannot be shared publicly because of [XXX]. Data are available from the XXX Institutional Data Access / Ethics Committee (contact via XXX) for researchers who meet the criteria for access to confidential data.*<br><br>*The data underlying the results presented in the study are available from (include the name of the third party*	All relevant data are within the manuscript and its Supporting Information files.

and contact information or URL).<br>• This text is appropriate if the data are owned by a third party and authors do not have permission to share the data.<br><br>* typeset	
Additional data availability information:	

Powered by Editorial Manager® and ProduXion Manager® from Aries Systems Corporation

Manuscript Click here to access/download;Manuscript;manuscript.doc

Study of the thermal regime of a reservoir on the Qinghai-Tibetan Plateau, China

Yanjing Yang [a], Yun Deng [a] *, Youcai Tuo [a], Jia Li [a], Tianfu He [a], Min Chen [a]

[a] State Key Laboratory of Hydraulics and Mountain River Engineering, Sichuan University, Chengdu 610065, China

E-mail address: 13281109119@163.com (Yanjing Yang); dengyun@scu.edu.cn (Yun Deng); tuoyoucai@scu.edu.cn (Youcai Tuo); lijia_scu@scu.edu.cn (Jia Li); 15802879611@163.com (Tianfu He); mchen@scu.edu.cn (Min Chen)

Abstract

The Qinghai-Tibetan Plateau region has unique meteorological characteristics with low temperature, low air pressure, low humidity, little precipitation, and strong diurnal variation. The features of reservoir stratification change with thermodynamic processes under different climate and hydrological conditions. To explore the typical thermal regime of stratified reservoirs on the Qinghai-Tibetan Plateau, we simulated water temperature structures in Pangduo Reservoir, which is a typical dimictic reservoir on the Qinghai-Tibetan Plateau, adopting a two-dimensional numerical model. Field data was used to verify the model, and results showed that the model accurately simulated the vertical water temperature structure through a year as well as the whole ice period during winter. During the heating and the high-temperature period among a year, thermocline depth showed an evident diurnal variation within 20 m below the surface, inducing the deepening of surface mixing layer in the reservoir. However, the characteristics of diurnal variation were not significant in winter due to the influence of ice cover. Compared to reservoirs in low-altitude areas at the same latitude, Pangduo Reservoir behaved a shorter stratification period and weaker stratification stability, and the unique shortwave radiation process was majorly responsible for that according to the heat budget analysis. This study provided a scientific understanding of the thermal changes in stratified reservoirs under the special geographical and meteorological conditions on the

Qinghai-Tibetan Plateau. Moreover, this model can serve as a reference for decisions related to the adaptive management of similar reservoirs on high-altitude areas.

Keywords: Qinghai-Tibetan Plateau; Reservoir thermal regime; Numerical modeling; Temperature diurnal variation

Introduction

When a river dam is used to regulate runoff, it will inevitably affect the spatial and temporal changes in water temperature in the reservoir area and downstream rivers [1]. The velocity of the shallow channel is fast, and the vertical mixing is basically uniform. However, the construction of the dam transforms the water into a relatively static or slow-flowing general state, forming unique reservoir temperature stratification characteristics [2,3]. The stratified thermal structure is the basic physical characteristic of the lake, which increases the risk of water quality deterioration, such as the reduction of nutrients and dissolved oxygen in deep water [4-6]. Moreover, the temperature stratification of reservoir water will affect the utilization of reservoir water, and this process likely has a negative influence on reservoir and river aquatic life in water ecosystems [7-9]. Research on the evolution law of the water thermal regime is the basis for optimizing the allocation of reservoir water and mitigating its negative effects [10,11]. Many countries have conducted several studies on the water temperature of reservoirs based on their specific conditions [12-16].

Surface mixed layers of reservoirs periodically appear and disappear with variations in heat flux [17]. The heat absorption of the water surface has an important influence on the heating process of the thermocline during the heating period. In the cooling period, the heat loss of the surface water drives vertical mixing, and the inflow of low-temperature water forms an intrusion flow, which jointly controls the cooling process of the thermocline [18,19]. Temperature, radiation and local winds are the most important factors in the thermal processes in lakes or reservoirs [20-22]. However, the impact of climate change is unique because the stratified characteristics of lakes (reservoirs) are affected by factors such as the density structures, morphological characteristics (deep

run-of-the-river and lake-type reservoirs), and artificial regulation of reservoirs [23-26]. Therefore, it is meaningful to explore the response to climate change and the thermal state of the reservoir.

In recent years, considerable efforts have been made to develop a set of highly indicative description systems combined with factors such as stratification time and thermocline characteristics to express the lake reservoir stratification response to changes in external factors [27,28]. The current research mainly focuses on the regularity of water temperature stratification in response to annual and seasonal changes [29,30]. However, related research indicates that the reservoir would have a more subtle mixing process within a day; specifically, the warm water layer is heated during the day and cooled at night and is subject to the influence of temperature fluctuation every day and night. Surface water always has a noticeable convection effect, which likely weakens the buoyancy effect of surface water [31-33]. Moreover, early research focused primarily on regional differences. The stratification of lakes and reservoirs is related to local meteorology, water temperature and geographical morphology; however, the lack of detailed data samples and perfect research methods reflect certain existing limitations [34].

With the development of China's water conservancy projects in the source region of the Qinghai-Tibetan Plateau, the evolution of lake and reservoir water temperature is becoming increasingly complicated [22]. The Qinghai-Tibetan Plateau has unique weather and atmospheric circulation characteristics, which have a significant impact on the Asian monsoon, global atmospheric circulation and global climate change [35,36]. The Qinghai-Tibetan Plateau is characterized by low temperature, low air pressure, low humidity, little precipitation, a simple ecosystem structure and weak anti-interference ability. In addition, human activities and global environmental changes can easily affect the anti-interference capability of the Qinghai-Tibetan Plateau, which shows strong ecological vulnerability [37-39]. Local aquatic organisms on the Qinghai-Tibetan Plateau grow slowly and are more sensitive to changes in hydraulic conditions such as water temperature and flow velocity. Thus, the impact of water temperature changes on their ecological environment will be more significant [40]. There have

been few investigations on the water temperature characteristics of reservoirs on the Qinghai-Tibetan Plateau. Previous studies have demonstrated that the epilimnion, metalimnion and hypolimnion layers could be obviously distinguished in Nam Co with different physicochemical features [41]. Moreover, few scientists seem to be aware of the thermal characteristics of reservoirs on the Qinghai-Tibetan Plateau, and the laws of changes in mixing in reservoir water bodies are still unclear.

To explore the thermal characteristics of stratified reservoirs in the special geographical and meteorological environment of the Qinghai-Tibetan Plateau, the Pangduo Reservoir in the southwestern Qinghai-Tibetan Plateau was used and assessed with a two-dimensional mathematical model of water temperature and hydrodynamics based on CE-QUAL-W2; furthermore, the model was validated with measured water temperature data from the study area. The validated mathematical model will be used to simulate the response of the thermal characteristics of the Qinghai-Tibetan Plateau Reservoir to changes in local climate and hydrological conditions and to reveal the thermal stability characteristics of the reservoir as well as the characteristics of the diurnal variation in the thermal state that is unique to the plateau reservoir.

The case study

Study area

The Qinghai-Tibetan Plateau is the highest geomorphic tectonic unit in the world, ranging from N 26°00' to N 39°47' and E 73°19' to E 104°47’. A unique hydrothermal condition has been formed due to the restriction of atmospheric circulation and plateau topography. The Pangduo Reservoir (N 30°51'; E 91°21') is located in the southwestern part of the Qinghai-Tibetan Plateau and is a controlling project on the Lhasa River. The total storage capacity of the reservoir is 12.3 × 10^8 m^3; the normal storage level is 4,095.0 m, and the dead water level is 4,072.0 m. The total length of backwater in the reservoir area is 46.51 km, the total water area is 43.7 × 10^6 m^3, and the

depth in front of the dam is 64.3 m. The installed capacity of the power station is 120 MW. The function of the Pangduo Reservoir is irrigation and power generation, and the reservoir also provides functions including flood control, urban water supply, and ecological environmental protection.

Fig. 1 Study area diagram.

Regional monitoring

To understand the thermal characteristics of this plateau reservoir and its relationship with meteorological responses, we monitored the water temperature and meteorological conditions of the Pangduo Reservoir from August 23, 2016, to September 28, 2017. The monitoring scheme is shown in Table 1. The monitoring points (Figs 1A1 and 1A2) were set up at the end of the Rezhenzangbu and Wululong tributary reservoirs, and the monitoring frequency was once per hour. A vertical water temperature automatic monitoring temperature chain was installed in front of the dam for short-term monitoring (Fig 1B1) from August 25 to August 31, 2016, and the monitoring frequency was once per hour; the vertical interval of sampling depth was 1 m. Manual on-site inspections and reviews were conducted monthly. Other factors, such as air temperature, ambient humidity, wind speed, wind direction, and solar radiation, were monitored by a self-recording weather station (Fig 1A3) installed on the top of the dam during the monitoring period.

Table 1. Monitoring scheme

Project	Point Location	Latitude and Longitude	Instrument	Period and Frequency
Continuous water temperature	A1: Wululong	91°12'19"E30°15'51"N	ZDR-20h	2016.08.25~2017.09.28, Once per hour.
	A2: Rezhenzangbu	91°31'17"E30°18'20"N		
Meteorological	A3: Dam top	91°21'49"E30°10'55"N	Jinzhou SunshinePC-4	
Vertical water temperature	B1: In front of dam	91°21'09"E30°11'15"N	TidbiT v2（UTBI-001）	2016.08.25~2016.08.31, Once per hour.

Manual monitoring	B1: In front of dam	91°21'09"E30°11'15"N	EXO2	2016.09~2016.12, Once a month

Satellite pictures in winter

The Pangduo Reservoir freezes in winter. To understand the growth, development, and ablation processes of winter ice in the Pangduo Reservoir, we used landsat8 satellite data to identify the classification of ice and water in the reservoir area based on the spectral differences of different features (S1 Fig). With the decrease in air and inflow water temperature in early December, affected by the water-gas heat exchange and the solid boundary heat conduction, the ice first appeared in the shallow water of the reservoir tail, with an ice coverage of 6%. In mid-February, as the water body continued to lose heat, the initial ice cover formed in the Wululong branch, the water surface was frozen, and the ice area of the Rezhenzangbu branch increased rapidly. The area in front of the dam was frozen, and the ice coverage rate in the reservoir area reached 67.35%. As the air and inflow water temperature increased by the end of March, the ice surface began to thaw, and the ice coverage rate in the reservoir area declined to 50.4%. Finally, the freezing period ended at the end of April.

Mathematical models and methods

According to the monitoring data, the air temperature was low in the study area. The radiation was strong, and the inflow water temperature and the air temperature had great diurnal variation. The vertical water temperature had clear characteristics of diurnal variation. To quantify the thermal conditions of the reservoirs on the Qinghai-Tibetan Plateau and the characteristics of their unique diurnal variation, CE-QUAL-W2, a two-dimensional hydrodynamics and water quality model, was used. This paper constructed a longitudinal and vertical two-dimensional water temperature model for the whole reservoir area for the research. This model ignored the variation in various variables along the width of the river; thus, the model could be applied to water bodies with longitudinal and vertical temperature gradients in the Pangduo Reservoir [42]. The main input boundaries of the

model include inflow, outflow, inflow water temperature, and meteorological conditions.

Governing equations

The governing equations include mass conservation and conservation of momentum using the Boussinesq approximation: the effects of density changes are considered only in the gravity term, and the hydrostatic pressure assumption is used [42].

Continuous equation:

$$\frac{\partial BU}{\partial x}+\frac{\partial BW}{\partial z}=(q+q_b)B \tag{1}$$

X-direction momentum equation:

$$\frac{\partial UB}{\partial t}+\frac{\partial UUB}{\partial x}+\frac{\partial WUB}{\partial z}=gB\sin\alpha+g\cos\alpha B\frac{\partial\eta}{\partial x}$$
$$-\frac{g\cos\alpha B}{\rho}\int_{\eta}^{z}\frac{\partial\rho}{\partial x}dz+\frac{1}{\rho}\frac{\partial B\tau_{xx}}{\partial x}+\frac{1}{\rho}\frac{\partial B\tau_{xz}}{\partial z}+q_b BU_x \tag{2}$$

$$U_x=U_b\cos\beta \tag{3}$$

Hydrostatic pressure assumption:

$$0=g\cos\alpha-\frac{1}{\rho}\frac{\partial p}{\partial z} \tag{4}$$

Free surface equation:

$$B_\eta\frac{\partial\eta}{\partial t}=\frac{\partial}{\partial x}\int_{\eta}^{h}Budz-\int_{\eta}^{h}qBdz \tag{5}$$

Heat transport equation:

$$\frac{\partial B\rho C_P T}{\partial t}+\frac{\partial UB\rho C_P T}{\partial x}+\frac{\partial WB\rho C_P T}{\partial z}$$
$$-\frac{\partial\left(BD_x\frac{\partial\rho C_P T}{\partial x}\right)}{\partial x}-\frac{\partial\left(BD_z\frac{\partial\rho C_P T}{\partial z}\right)}{\partial z}=S_\Phi B \tag{6}$$

where B is the width of the water body, U is the longitudinal flow velocity, W is the vertical flow velocity, q is

the lateral unit length of the incoming flow, η is the water level, α is the river dip, ρ is the water density, and U_x is the x component of the tributary flow rate. U_b is the longitudinal flow velocity of the tributary, β is the angle of the dry tributary, q_b is the unit length of the tributary, $B\eta$ is the water surface width, D_x and D_z are longitudinal and vertical dispersion coefficients, respectively, and S_Φ is the atmospheric heat source term.

The water-air interface heat exchange is considered in the model

$$\varphi_{GY} = \varphi_{sn} + \varphi_{an} - \varphi_{br} - \varphi_{\varepsilon} + \varphi_c \quad (7)$$

where φ_{GY} is the plateau water-air heat exchange flux; φ_{sn} is the solar shortwave radiant heat flux; φ_{an} is the atmospheric longwave radiant heat flux; φ_{br} is the back radiant heat flux by water; φ_{ε} is evaporative heat flux; and φ_c is the heat conduction flux.

The ice cover in the model is as follows:

$$\rho_i L_f \frac{\Delta h}{\Delta t} = h_{ai}(T_i - T_e) - h_{wi}(T_w - T_m) \quad (8)$$

where ρ is the ice density, L_f is the latent heat of freezing, $\Delta h/\Delta t$ is the ice thickness growth rate, h_{ai} and h_{wi} are the heat exchange coefficients of ice-gas and ice-water, respectively, T_m is the temperature of the ice-water interface, T_e is the temperature at which the heat exchange between ice and air reaches equilibrium, T_w is the water temperature under ice, and T_i is the ice temperature.

The longitudinal eddy viscosity coefficient and the longitudinal eddy current diffusion coefficient both had default values of 1 m^2/s; the vertical eddy viscosity coefficient was calculated by the mixed length empirical formula W2N, which considers the temperature effect.

Model grid and calculation conditions

The Punto Reservoir area was divided into 131 × 37 (longitudinal × vertical) rectangular cells. The longitudinal size of the cell grid was 100 ~ 300 m, and the vertical size was 2 m.

The verification period was from August 25, 2016, to December 31, 2016. The temperature field at the initial

moment was obtained after interpolation based on the vertical water temperature measured in the reservoir area on August 25, 2016. Inflow water temperature and meteorological data used measured data. Water level, outflow and inflow used measured data from Pangduo Hydrological Station.

The calculation period was from August 25, 2016, to August 24, 2017. Inflow water temperature and meteorological data used measured data. The reservoir operation data adopted the designed operating conditions of the Pangduo Hydropower Station (S2 Fig).

Thermal stratification evaluation index

We selected indicators to quantify the thermal stratification of water bodies and evaluate the characteristics of the stratified structure of water temperature. The vertical water temperature gradient (*VTG*, °C/m) and the buoyancy frequency (*N*, 1/s) were calculated based on the water temperature calculation results of the vertical section in front of the dam.

$$VTG = \frac{\partial T(z)}{\partial z} \qquad (9)$$

$$N = \sqrt{-\frac{g}{\rho_0}\frac{\partial \rho(z)}{\partial z}} \qquad (10)$$

where $T(z)$ is the water temperature at depth z, °C. $\rho(z)$ is the density at depth z, kg/m^3, which is temperature dependent [42]. ρ_0 is the average density of the whole water column, kg/m^3. *g* is the acceleration of gravity, m/s^2. Although *N* is the convention used in limnology and oceanography, reservoirs are similar. Thus, the change trend of *N* can be used to evaluate the stratified stability of water columns. *N* indicates that in a stable temperature layered structure, the fluid particles move in the vertical direction after being disturbed. The combined effect of gravity and buoyancy always returns them to an equilibrium position and oscillates due to inertia. The oscillation frequency can be understood as the exchange rate of water [43].

To better evaluate the changes in the stratification stability of the water column, the *SI* index, which reflects

the stability of the deep water body, was used to evaluate the strength of the stratification stability of the reservoir [28].

$$SI = \sum_{Z=Z_0}^{Z=Z_l} (Z - \bar{Z})\rho_Z \qquad (11)$$

Here, Z is the depth of the water column from the surface, and Z_0, Z_l and $\bar{Z}$ are the depths of the surface water, the lower end of the water column, and the centroid of the water column, respectively. The water density at depth Z is ρ_Z. *SI* can be converted into energy by multiplying by the acceleration of gravity and the volume of each layer, and it represents the ideal energy estimate of the entire water column to achieve mixing in the depth range without an increase or decrease in heat.

Setting the zero-order decay rate to –1 day^{-1} and zeroing out all other generic constituent kinetic parameters results in a state variable that increases by 1 day^{-1}, which is an exact representation of water age or hydraulic residence time [42].

Results and discussion

Monitoring data analysis

The average air temperature (the mean was 6.8°C) of Pangduo was relatively low during the year (Fig 2A). The average monthly temperature varied from -2.1 to 13.7°C, with the lowest temperature in January and the highest temperature in July. The temperature varied greatly during the day, with an average of 11.2°C. The lowest temperature appeared from 6:00 to 7:00 a.m. during the day, and the highest temperature was from 3:00 to 4:00 p.m. The solar radiation in the monitoring area was strong (Fig 2B), and the average was 231.2 W/m^2 during the monitoring period. Atmospheric longwave radiation showed an upward trend from December to August of the following year and reached the highest value in August, at 359 W/m^2. The solar radiation was the strongest during the day from 12:00 to 2:00 p.m., with an average value of 980.4 W/m^2.

The inflow water temperature was low. The average water temperatures of the Rezhenzangbu (Fig 2C) and Wululong branches (Fig 2D) were 6.1°C and 6.7°C, respectively. The water temperature of the natural river entering the reservoir was mainly affected by meteorological conditions. During the monitoring period, the average daily variation in the temperature of the Rezhenzangbu tributary and Wululong tributary was 2.1°C and 3.2°C, respectively.

Fig. 2 Temporal distribution of measured air temperature, solar radiation, and inflow water temperature in the study area.

Fig 3 shows the distribution of the measured vertical water temperature in front of the dam from August 25 to August 31, 2016. There was significant diurnal variation in the vertical water temperature distribution within a range of 10-20 m from the surface of the reservoir area during the monitoring period. The typical study layers include the surface layer and layers 1, 2, 3, 4, 5, 10, 20, and 30 m below the surface. The daily variation in water temperature is shown in Table 2. The diurnal amplitude gradually decreased as the depth increased. The average daily variation in the surface layer over the 7 days was 4.1°C, with a range of 2.3°C ~ 5.7°C. The average daily variation was 0.7°C at a depth of 5 m below the surface layer, and the variation range was 0.4°C ~ 1.2°C. The average daily variation was 0.3°C at a depth of 30 m below the surface layer, which was only approximately 7.3% the value of the surface layer.

Fig. 3 Measured vertical water temperature changes from August 25, 2016, to August 31, 2016.

Table 2. Water temperature variation

Date	Air temperature variation /°C	Inflow temperature variation/°C	Water temperature variation/°C								
			0	1	2	3	4	5	10	20	30
2016/8/25	12.3	4.8	5.7	2.6	0.9	0.9	0.8	0.8	0.7	0.5	0.3
2016/8/26	9.6	4.5	3.9	1.5	1.2	1.0	0.6	0.5	1.1	0.3	0.3
2016/8/27	13.4	5.5	5.5	1.4	0.9	0.7	0.5	0.4	0.6	0.5	0.4
2016/8/28	12.6	4.9	5.7	3.0	2.5	1.3	0.8	0.5	0.4	0.4	0.4
2016/8/29	10.3	5.1	3.4	1.4	1.3	1.3	1.0	0.9	1.1	0.4	0.4

2016/8/30	6.8	5.2	2.3	0.9	0.9	1.1	1.2	1.2	0.5	0.4	0.3
2016/8/31	10.5	4.7	2.6	1.1	1.0	1.2	1.3	0.8	0.5	0.3	0.3
Average	10.8	5.0	4.1	1.7	1.3	1.1	0.9	0.7	0.7	0.4	0.3

The model calibration and validation

Fig 4 compares the measured and calculated results of the vertical water temperature in the reservoir area during each month during the verification period. The results showed that the temperature stratification of the reservoir area was weak. The surface water temperature decreased from 13.9°C in early September to 4.6°C in December. Comparing the calculated results with the actual measured results, the temperature variation trends of the epilimnion and metalimnion were consistent, and the temperature of the hypolimnion was generally well matched. The average error during the entire verification period was 0.1°C, the relative error was 1.3%, the standard deviation (STD) was 0.507, and the root-mean-square error (RMSE) was 0.189°C. The scatter plot of the measured and calculated water temperature showed that the degree of dispersion of the error was small (S3 Fig). The results showed that the model can accurately simulate the influence of buoyancy flow and atmospheric heat exchange on the stratified structure of water temperature in the reservoir.

Fig. 4 Comparison of calculated and measured water temperature in front of the dam.

The ice variation was also considered in the calculations. The calculated results showed that the ice in front of the dam began to appear in late December, with an average ice thickness of 0.11 m, and the maximum ice thickness appeared in mid-January, with a thickness of 0.35 m; the ice cover melted in late April. The results showed that the growth, development and ablation of the ice were in good agreement with the interpretation time obtained from the satellite images. The results showed that the two-dimensional mathematical model of water temperature was reliable.

Fig. 5 The variation process of the calculated ice thickness in front of the dam.

Thermal structure characteristics

Fig 6 shows the two-dimensional distribution process of the temperature (A), temperature gradient (B), buoyancy frequency (C), and water age (D) in front of the dam from August 25, 2016, to August 24, 2017. The results showed that the Pangduo Reservoir was a typical dimictic reservoir. The reservoir has two inversions: one at the end of March and one at the end of October in autumn. After each major inversion, the reservoir area will maintain the same vertical temperature for a period of time.

From September to November 2016, the reservoir was in a cooling period. The surface water temperature decreased from 14.7°C to 7.2°C, and the vertical temperature difference of the reservoir decreased from 4.0°C to 0.2°C. The position of the thermocline mainly existed within 20 m of the surface layer, and the vertical temperature gradient (VTG) changed within 0.3°C/m, with an average of 0.08°C/m. The buoyancy frequency varied between 0.1 H_z and 0.6 H_z, presenting a continuous unstable state, which was believed to be a diurnal thermocline phenomenon caused by the special meteorological and incoming water temperature changes on the Qinghai-Tibetan Plateau. The water body in the reservoir was mainly replaced in the upper and middle layers. Its water age varied between 20 and 60 days. The retention time of the water at the bottom of the reservoir was relatively long, at approximately 140 ~ 200 days; however, as the temperature of the inflow water decreased and the flow increased during the cooling period, vertical convection increased. At the end of October, the old water at the bottom of the reservoir was replaced and lifted.

Beginning in early December, the average daily temperature in the Pangduo area changed to a negative value, and the water temperature in the reservoir began trending to a vertical mixing until the temperature decreased to 4°C. When water continued to loss heat, the surface water cooled and sank [44]. As the surface water temperature decreased to 0°C, it was further supercooled, and a phase change occurred. Thin ice began to appear on the surface of the reservoir, forming an initial ice cap. The existence of ice caps transformed the heat exchange mode into the

heat exchange between the surface water body and the bottom of the ice cap and the heat exchange between the atmosphere and the upper surface of the ice cap [45]. An inverted structure of the vertical water body appeared. The temperature of the surface water body of the reservoir was approximately 0.5°C, and the temperature of the bottom of the reservoir was maintained at 4.0°C. Near the intake, the buoyancy frequency varied from 0.2 H_z to 0.3 H_z. With the decrease in the flow, the retention of the water body increased and advanced evenly to the front of the dam, changing between 120 and 180 days.

In May 2017, as the air and inflow water temperature increased, the temperature of the surface water also increased. The water body of a certain thickness on the surface layer began to convect and then reached the same temperature as that of the whole reservoir, approximately 4.0°C. By the end of May, the surface water temperature continued to increase to 7.4°C. Water temperature stratification began to occur in the reservoir from June to August. The surface water temperature increased to 13.8°C by the end of August, and the vertical temperature difference expanded to 6.5°C. At this time, the buoyancy frequency variation range of the surface layer in the range of 20 m expanded to 0.1 H_z to 0.6 H_z, and a diurnal thermocline appeared. With the increase in the flow and the buoyancy, the replacement of the surface water body began to increase, changing between 20 and 60 days. The water temperature at the bottom of the reservoir was low, the water density was high, and it was not easy for the water to mix and exchange with the upper water body. The water age began to increase, up to 160 days. Due to the weak stratification of the reservoir area and the existence of a diurnal thermocline, the replacement of the upper and middle water bodies occurred sooner. Compared with that of the reservoirs and lakes on the low-altitude areas, the retention time was relatively short [46,47].

Fig. 6 Vertical water temperature (A), gradient (B), buoyancy frequency (C), and water age (D) distribution in the calculation period of the Pangduo Reservoir.

Diurnal characteristics analysis

To explore the characteristics of diurnal variations in reservoir water temperature during the high-temperature period, the simulation results on August 15, 2017, were selected as a representative day (Fig 7). On August 15, the air temperature fluctuated from 10.3°C to 20.7°C, with the lowest temperature at 7:00 a.m. and the highest temperature at 4:00 p.m.

The simulated results showed that the characteristics of the diurnal changes in front of the dam were significant. The surface water temperature decreased to a minimum of 14.0°C at 8:00 a.m., the buoyancy frequency (Fig 8B) within a range of 20 m changed between 0.4 H_z and 0.6 H_z, and the vertical temperature difference was 1.2°C. At 4:00 p.m., the surface water temperature increased to 15.1°C, the average buoyancy frequency of 5 m on the surface layer was 0.4 H_z, and the vertical temperature difference within 20 m increased to 2.5°C. This phenomenon occurred because the average net heat flux (Fig 8A) was -164.17 W/m^2 during the period from 0:00 a.m. to 8:00 a.m. The surface water column lost heat and cooled, the density increased, the water column gradually mixed downward, and the water exchange rate increased. From 9:00 a.m. to 7:00 p.m., the average net heat flux of the surface water layer was 280.76 W/m^2. The surface water column absorbed heat and warmed, and the buoyancy of surface water increased, which inhibited the vertical mixing of the water column. Only the diffusion effect was used to transfer heat to the lower water layers, and there was an obvious vertical temperature gradient. From 7:00 p.m. to 11:00 p.m., the average net heat flux of the surface water body was -129.00 W/m^2, and the water body began to lose heat. This intraday thermal expansion and contraction will cause the mechanical disintegration of unstable structures due to gravity, leaving the surface water body in an unstable motion state [32].

The diurnal variation in the tail section of the reservoir was obvious. The inflow changed over the entire depth range, and the daily temperature differences at the surface layer and the bottom layer were both 1.1°C, which was very unique. The mixing mode was mainly controlled by the influent water temperature change (Fig 8A). The

temperature of the inflow water fluctuated from 11.0°C to 15.6°C, with a daily change of 4.6°C. From 0:00 a.m. to 8:00 a.m., the average inflow water temperature was 13.8°C, and the inflow water invaded the reservoir horizontally. From 9:00 a.m. to 7:00 p.m., the average water temperature of the inflow affected by glacial recharge decreased to 12.4°C, the density of the water body increased, and a subsurface current gradually formed. From 8:00 p.m. to 11:00 p.m., the temperature of the inflow water increased, with an average value of 15.1°C. The density of the inflow water decreased, leaving the river bottom and transitioning to the surface. When the water flowed into the mid-reservoir, it was affected by the temperature stratified anisotropic buoyancy flow, and the inflow water temperature no longer dominated.

Fig. 7 Longitudinal and vertical two-dimensional water temperature and flow field distribution in the Pangduo Reservoir on August 15, 2017.

Fig. 8 Diurnal distribution of net heat flux in front of the dam and inflow temperature (A) and buoyancy frequency in front of the dam (B) on August 15, 2017.

To explore the characteristics of diurnal variations in reservoir water temperature in winter, the simulation results on February 15, 2017, were selected as a representative day (Fig 9).

The simulated results showed that the diurnal characteristics of winter were not obvious. The temperature of the surface water in front of the dam ranged from 2.1°C to 2.3°C, and the temperature at the bottom of the reservoir was stable at 4.0°C. The reservoir area was completely frozen in February, and the ice layer provided thermal protection for the water body such that the heat exchange method of the surface water layer changed from "water-air heat exchange" to "ice-water heat exchange" [48,49]. The inflow water at the tail of the reservoir was close to 0°C, and it was gradually pushed towards the reservoir along the bottom of the ice cover due to the buoyancy effect of the cold water. The water temperature structure of the Pangduo Reservoir remained inverted throughout the ice-sealing period with small differences.

Fig. 9 Longitudinal and vertical two-dimensional water temperature and flow field distribution in the Pangduo Reservoir on February 15, 2017.

Comparison with the low-altitude reservoirs at the same latitude

To more clearly compare the thermal conditions between the reservoir on the Qinghai-Tibetan Plateau and those in the low- and medium-altitude regions, the same mathematical model was used to replace the water temperature and meteorological boundary of the reservoirs in the low-altitude area with an average altitude of 180 m at the same latitude (Fig 11A). During the calculation period, the average air temperature and inflow water temperature were higher (20.9°C and 19.9°C, respectively), and the solar radiation was lower (118.79 W/m^2), which was significantly different from that of the Qinghai-Tibetan Plateau.

The water temperature of the reservoir in the low-altitude area was always greater than 4°C, indicated that it is a typical monomictic reservoir (Fig 11B). From September to December 2016, the surface water temperature decreased from 28.3°C to 17.0°C, and the vertical temperature difference narrowed from 17.8°C to 8.6°C. By the winter of February 2017, the surface water temperature had decreased to 13.8°C, with the vertical temperature difference had narrowed to 4.3°C. At the beginning of April, the surface water temperature began to rise, increasing to 31.0°C by August, and the vertical temperature difference widened to 21.6°C.

The continuous decrease in air temperature during the cooling period cooled the surface water temperature, and the density of the surface water body increased. The sinking convection effect gradually deepened from the surface layer first. In winter, the water column was vertically flipped and mixed within a depth of 40 m in front of the dam. Due to the deep depth, the water body at the bottom of the reservoir was not completely replaced. During the warming period, the reservoir formed stable density stratification. Similar to most middle-low-altitude stratified reservoirs, the three-layer structure of the epilimnion, metalimnion and hypolimnion was maintained in front of the dam [50,51].

Fig 10 indicates the comparison of daily average heat budget of surface water between the Pangduo Reservoir and the low-altitude reservoir during the calculated period. From September to December 2016, the average net heat flux of the Pangduo Reservoir and the low-altitude reservoir was -57.38 W/m^2 and -50.84 W/m^2, respectively. Before the winter of April 2017, the Pangduo Reservoir was frozen, and the heat exchange method of the surface water layer changed from "water-air heat exchange" to "ice-water heat exchange". The average heat loss of the surface water was slight and stable (the mean was -25.27 W/m^2). At the same time, the net heat flux was fluctuating and ranged from -160.80 W/m^2 to 154.37 W/m^2 in the low-altitude reservoir. From the end of April to August, the average net heat flux in the Pangduo Reservoir and the low-altitude reservoir were 37.11 W/m^2 and 35.12 W/m^2, respectively.

Fig. 10 Comparison of daily average heat budget of surface water between the Pangduo Reservoir (A) and the low-altitude reservoir (B) during the calculated period.

Fig 11C shows a comparison of the *SI* changes between the Pangduo Reservoir and the low-altitude reservoir. The average annual *SI* of the low-altitude reservoir was 353.75 kg/m^2, and the annual variation range was 43.03~712.68 kg/m^2. From September to December, it showed a downward trend, with an average of 377.68 kg/m^2 during the period. The average *SI* from January to March was 65.78 kg/m^2. The *SI* value increased from April to June, with an average of 407.04 kg/m^2. During the high-temperature period in summer, the *SI* remained high in July and August, with an average value of 657.93 kg/m^2. However, the average annual *SI* of the Pangduo Reservoir was 26.4 kg/m^2, which was 7.46% that of the low-altitude reservoir. The annual variation range was 0 ~ 93.13 kg/m^2, which was 13.91% that of the low-altitude reservoir. From November to May of the following year, the *SI* was close to 0 kg/m^2. The average *SI* from June to August was 76.10 kg/m^2. Compared with the reservoir at the same latitude in the low-altitude area with the same regulation performance and scale, the reservoir on the Qinghai-Tibetan Plateau had shorter stratification periods and weaker stratification intensities.

Fig. 11 Air temperature and inflow water temperature distribution of the low-altitude reservoir during the calculated period (A). Comparison of vertical water temperature between the low-altitude reservoir and the Pangduo Reservoir in front of the dam during the calculation period (B). Comparison of SI changes between the Pangduo Reservoir and the low-altitude reservoir (C).

Fig 12 shows the comparison of diurnal heat budget of surface water between the low-altitude reservoir and the Pangduo Reservoir on August 15, 2017. The epilimnion was affected by diurnal temperature fluctuations, heating during the daytime and cooling at night [52]. The net radiation of Pangduo fluctuated drastically with a diurnal variation of 945.76 W/m^2, which was positive between 9:00 a.m. and 7:00 p.m. and reached the highest at 1:00 p.m., and the diurnal variation in the low-altitude reservoir was 71.8% that of Pangduo. There was always a noticeable temperature increase and stratification on the surface during sunlight hours [53]. Whether in the Pangduo reservoir or the low-altitude reservoir, shortwave radiation had a greater impact on the net heat flux, which affected the trend and inflection point of the net heat flux. The shortwave radiation of the Pangduo reservoir was strong, and the highest was 898.34 W/m^2 at 1:00 p.m., while the highest shortwave radiation of the low-altitude reservoir was 542.92 W/m^2 at 11:00 a.m. The net longwave radiation of the Pangduo Reservoir was generally smaller than that of the low-altitude reservoir, with an average daily difference of 35.5 W/m^2. Evaporation heat flux accounted for a large proportion of heat loss, which in the Pangduo Reservoir was higher than that of the low-altitude reservoir, with average values of 125.58 W/m^2 in the Pangduo Reservoir, with 29.3% higher than that of the low-altitude reservoir. Heat conduction was slight, whether in the Pangduo Reservoir or the low-altitude reservoir. Generally, they caused differences in the amplitude and phase of the net heat flux.

Fig. 12 Comparison of diurnal heat budget of surface water between the Pangduo Reservoir (A) and the low-altitude reservoir (B) on August 15, 2017.

Fig 13 shows the comparison of the daily variation in the buoyancy frequency of the water column in each layer in front of the dam in the low-altitude reservoir and the Pangduo Reservoir during the high-temperature

period on August 15, 2017. Compared with the Pangduo Reservoir, the daily variation characteristics of the low-altitude reservoir were not obvious. The average buoyancy frequency difference of the low-altitude reservoir was 0.13 H_z, and that of the Pangduo reservoir was 0.18 H_z. The temperature fluctuation range of the low-altitude reservoir was 26.0°C~34°C, and the surface water temperature varied within the range of 30.4°C~31.7°C. However, this diurnal variation stayed within only 5 m of the surface layer, and the average diurnal buoyancy frequency difference was 0.59 H_z. The reason was that the radiant heat loss from the surface layer at night was inadequate to transform sufficient potential energy into turbulent kinetic energy, as in the Qinghai-Tibetan Plateau reservoir, thereby forming a turbulent penetrating convection effect. Similar to most low-altitude reservoirs, it still showed stable buoyant flow [54,55]. At the same time, the inflow water temperature in the low-altitude reservoir was stable and varied between 28.0°C and 28.3°C. The inflow steadily entered the mainstream layer along the subduction line, which was significantly different from the special intraday inflow mixing model of the Qinghai-Tibetan Plateau.

Fig. 13 Comparison of the daily variation in the buoyancy frequency of the water column in each layer in front of the dam in the low-altitude reservoir and the Pangduo Reservoir.

Conclusion

(1) The reservoir of the Qinghai-Tibetan Plateau is a typical dimictic reservoir, and the reservoir had two inversions: one at the end of March and one at the end of October. In the summer, the warm surface water varied between 13.0°C~ 15.0°C, and it floated on the cold bottom water of 4.0°C ~ 7.0°C. The thermal regime of the reservoir changed with the cooling surface water in autumn and the overall inversion in winter. The vertical temperature difference gradually decreased to the same temperature. The ice cover in winter provided thermal protection for the water body, and the water body appeared to have an inverse temperature structure. The surface water temperature of the reservoir was approximately 0.5°C, and the bottom temperature was maintained at 4.0°C. During the warming period, as the air and inflowing water temperature increased, the temperature of the surface

layer also increased. The upper and middle water bodies began to convect and then reached the same temperature as that of the whole reservoir. Temperature stratification occurred from June to August. The strong diurnal variation in the meteorology and the inflow water temperature strengthened the vertical mixing of the surface water body of the reservoir. A diurnal thermocline appeared, and the reservoir water temperature was weakly stratified.

(2) Affected by the great diurnal changes in meteorology and inflow, the diurnal characteristics of water temperature in the Qinghai-Tibetan Plateau reservoir were obvious. The vertical temperature difference within 20 m in front of the dam varied between 1.2°C ~ 2.5°C, and the buoyancy frequency varied from 0.1 Hz to 0.6 Hz. In the early morning, the surface layer lost heat and cooled, with increasing density gradually blending downward. During the daylight hours, the surface water absorbed heat and enhanced buoyancy, which suppressed vertical mixing of the water column. This thermal expansion and contraction caused the surface water layers to remain in an unstable motion state. The tail section of the reservoir was affected by the diurnal variation in the inflow water temperature, and there was a unique intraday inflow mixing mode. The inflow varied across the entire depth range. In the early morning, the inflow temperature was relatively high, and the water invaded the reservoir horizontally. Under the influence of glacial replenishment during daylight, the inflow temperature water decreased, and a subsurface current gradually formed. At night, as inflow temperature increased, the water gradually flowed to the surface. In winter, the diurnal variation was not obvious due to the thermal protection of the ice cover.

(3) Compared with the monomictic low-altitude reservoirs at the same latitude, which had the same regulating performance and scale, the annual average *SI* value of the Pangduo Reservoir was 26.4 kg/m^2, which was only 7.5% that of the low-altitude reservoir. The annual variation range of the *SI* value was 0~93.13 kg/m^2 in the Pangduo Reservoir, which was 13.9% that of the low-altitude reservoir. In contrast, the Pangduo Reservoir had a shorter stratification period and weaker stratification intensity than that of the low-altitude reservoir.

Acknowledgments

This work was supported by the National Key R&D Program of China (grant number2016YFC0502202) and the National Natural Science Foundation of China (grant numbers 91547211).

References:

1. Alavian V, Jirka GH, Denton RA, Johnson MC, Stefan HG (1992) Density currents entering lakes and reservoirs. Journal of Hydraulic Engineering 118: 1464-1489. https://doi.org/10.1061/(ASCE)0733-9429(1992)118:11(1464)

2. Wetzel RG (1983) Limnology, Saunders College Publ. Philadelphia, PA: 207208.

3. Ford DE, Stefan H (1980) Stratification variability in three morphometrically different lake under identical meteorological forcing. JAWRA Journal of the American Water Resources Association 16(2), 243-247. https://doi.org/10.1111/j.1752-1688.1980.tb02385.x

4. Becker V, Caputo L, Ordóñez J, Marcé R, Armengol J, et al. (2010) Driving factors of the phytoplankton functional groups in a deep Mediterranean reservoir. Water research 44: 3345-3354. https://doi.org/10.1016/j.watres.2010.03.018

5. Wells SA (2000) Hydrodynamic and water quality river basin modeling using CE-QUAL-W2 version 3. WIT Transactions on Ecology and the Environment 41.

6. Tierney JE, Mayes M, Meyer N, Johnson C, Swarzenski PW, et al. (2010) Late-twentieth-century warming in Lake Tanganyika unprecedented since AD 500. Nature Geoscience 3: 422-425. https://doi.org/10.1038/ngeo865

7. Martin JL (1988) Application of two-dimensional water quality model. Journal of Environmental Engineering 114: 317-336. https://doi.org/10.1061/(ASCE)0733-9372(1988)114:2(317)

8. Verburg P, Hecky RE, Kling H (2003) Ecological consequences of a century of warming in Lake Tanganyika. Science 301: 505-507. https://doi.org/10.1126/science.1084846

9. Labaj AL, Michelutti N, Smol JP (2016) Changes in cladoceran assemblages from tropical high mountain lakes during periods of recent climate change. Journal of Plankton Research 39: 211-219. https://doi.org/10.1093/plankt/fbw092

10. Owens EM, Effler SW, Trama F (1986) Variability in thermal stratification in a reservoir. JAWRA Journal of the American Water Resources Association 22(2), 219-227. https://doi.org/10.1111/j.1752-1688.1986.tb01878.x

11. Rounds SA. Modeling water quality in the Tualatin River: achievements and limitations; 2001 2001-01-01. Citeseer. pp. 115-120.

12. Vermeyen TB. Use of temperature control curtains to modify reservoir release temperatures; 1995 1995-01-01. ASCE. pp. 521-525.

13. Lewis WM, Mccutchan JH, Roberson J (2019) Effects of Climatic Change on Temperature and Thermal Structure of a Mountain Reservoir. Water Resources Research 55: 1988-1999. https://doi.org/10.1029/2018WR023555

14. Li Y, Huang T, Zhou Z, Long S, Zhang H (2017) Effects of reservoir operation and climate change on thermal stratification of a canyon-shaped reservoir, in northwest China. Water Science & Technology: Water Supply 18: 418-429. https://doi.org/10.2166/ws.2017.068

15. Weber M, Rinke K, Hipsey MR, Boehrer B (2017) Optimizing withdrawal from drinking water reservoirs to reduce downstream temperature pollution and reservoir hypoxia. Journal of environmental management 197: 96-105. https://doi.org/10.1016/j.jenvman.2017.03.020

16. Fischer HB, List JE, Koh CR, Imberger J, Brooks NH (2013) Mixing in inland and coastal waters: Elsevier. https://doi.org/10.1016/B978-0-08-051177-1.50005-2

17. Zhang Y, Wu Z, Liu M, He J, Shi K, et al. (2015) Dissolved oxygen stratification and response to thermal structure and long-term climate change in a large and deep subtropical reservoir (Lake Qiandaohu, China). Water Research 75: 249-258. https://doi.org/10.1016/j.watres.2015.02.052

18. He W, Lian J, Zhang J, Yu X, Chen S (2019) Impact of intra-annual runoff uniformity and global warming on the thermal regime of a large reservoir. Science of The Total Environment 658: 1085-1097. https://doi.org/10.1016/j.scitotenv.2018.12.207

19. Xie Q, Liu Z, Chen Y, Chen X (2019) Internal waves in a deep run-of-river reservoir under hydro-peaking. Journal of Hydroelectric Engineering 38: 41-51. https://doi.org/10.11660/slfdxb.20190105

20. Zhang Y, Wu Z, Liu M, He J, Shi K, et al. (2014) Thermal structure and response to long‐term climatic changes in Lake Qiandaohu, a deep subtropical reservoir in China. Limnology and Oceanography 59: 1193-1202. https://doi.org/10.4319/lo.2014.59.4.1193

21. Saber A, James DE, Hayes DF (2018) Effects of seasonal fluctuations of surface heat flux and wind stress on mixing and vertical diffusivity of water column in deep lakes. Advances in water resources 119: 150-163. https://doi.org/10.1016/j.advwatres.2018.07.006

22. Huang L, Wang J, Zhu L, Ju J, Daut G (2017) The warming of large lakes on the Tibetan Plateau: Evidence from a lake model simulation of Nam Co, China, during 1979‐2012. Journal of Geophysical Research: Atmospheres 122: 13-95. https://doi.org/10.1002/2017JD027379

23. Magee MR, Wu CH (2017) Response of water temperatures and stratification to changing climate in three lakes with different morphometry. Hydrology and Earth System Sciences 21: 6253-6274. https://doi.org/10.5194/hess-2016-262

24. Butcher JB, Nover D, Johnson TE, Clark CM (2015) Sensitivity of lake thermal and mixing dynamics to climate change. Climatic Change 129: 295-305. https://doi.org/10.1007/s10584-015-1326-1

25. Lerman A, Imboden DM, Gat JR, Chou L (1995) Physics and chemistry of lakes: Springer-Verlag Berlin. https://doi.org/10.1002/iroh.19960810312

26. Norton GE, Bradford A (2009) Comparison of two stream temperature models and evaluation of potential management alternatives for the Speed River, Southern Ontario. Journal of Environmental Management 90: 866-878. https://doi.org/10.1016/j.jenvman.2008.02.002

27. Liu M, Zhang Y, Shi K, Zhu G, Wu Z, et al. (2019) Thermal stratification dynamics in a large and deep subtropical reservoir revealed by high-frequency buoy data. Science of the Total Environment 651: 614-624. https://doi.org/10.1016/j.scitotenv.2018.09.215

28. Sahoo GB, Forrest AL, Schladow SG, Reuter JE, Coats R, et al. (2016) Climate change impacts on lake thermal dynamics and ecosystem vulnerabilities. Limnology and Oceanography 61: 496-507. https://doi.org/10.1002/lno.10228

29. Jan-Tai K, Wu J, Wen-sen C (1994) Water quality simulation of Te-Chi Reservoir using two-dimensional models. Water Science and technology 30: 63. https://doi.org/10.2166/wst.1994.0029

30. Tuo Y, Li J, Deng Y, Liang R. Effects of water temperature on spawning ground of Chinese Sturgeon in Three Gorges Reservoir; 2011 2011-01-01. IEEE. pp. 5916-5919. https://doi.org/10.1016/j.coldregions.2017.08.014

31. Vercauteren N, Huwald H, Bou Zeid E, Selker JS, Lemmin U, et al. (2011) Evolution of superficial lake water temperature profile under diurnal radiative forcing. Water resources research 47. https://doi.org/10.1029/2011WR010529

32. MacIntyre S, Romero JR, Kling GW (2002) Spatial‐temporal variability in surface layer deepening and lateral advection in an embayment of Lake Victoria, East Africa. Limnology and oceanography 47: 656-671. https://doi.org/10.4319/lo.2002.47.3.0656

33. Van Emmerik T, Rimmer A, Lechinsky Y, Wenker K, Nussboim S, et al. (2013) Measuring heat balance residual at lake surface using Distributed Temperature Sensing. Limnology and Oceanography: Methods 11: 79-90. https://doi.org/10.4319/lom.2013.11.79

34. Kraemer BM, Anneville O, Chandra S, Dix M, Kuusisto E, et al. (2015) Morphometry and average temperature affect

lake stratification responses to climate change. Geophysical Research Letters 42: 4981-4988. https://doi.org/10.1002/2015GL064097
35. Ding Y, Ye B, Han T, Shen Y, Liu S (2007) Regional difference of annual precipitation and discharge variation over west China during the last 50 years. Science in China Series D: Earth Sciences 50: 936-945. https://doi.org/10.1007/s11430-007-0042-8
36. You Q, Kang S, Wu Y, Yan Y (2007) Climate change over the Yarlung Zangbo river basin during 1961–2005. Journal of Geographical Sciences 17: 409-420. https://doi.org/10.1007/s11442-007-0409-y
37. Wang XD, Zhong XH, Liu SZ, Liu JG, Wang ZY, et al. (2008) Regional assessment of environmental vulnerability in the Tibetan Plateau: Development and application of a new method. Journal of Arid environments 72: 1929-1939. https://doi.org/10.1016/j.jaridenv.2008.06.005
38. Li XY, Ma YJ, Xu HY, Wang JH, Zhang DS (2009) Impact of land use and land cover change on environmental degradation in Lake Qinghai watershed, northeast Qinghai - Tibet Plateau. Land Degradation & Development 20: 69-83. https://doi.org/10.1002/ldr.885
39. Zhu L, Wang J, Ju J, Ma N, Zhang Y, et al. (2019) Climatic and lake environmental changes in the Serling Co region of Tibet over a variety of timescales. Science Bulletin 64: 422-424. https://doi.org/10.1016/j.scib.2019.02.016
40. Xu M, Wang Z, Pan B, Gong T, Liu L (2012) Research on assemblage characteristics of macroinvertebrates in the Yalu Tsangpo River Basin. Shengtai Xuebao/Acta Ecologica Sinica 32: 2351-2360. https://doi.org/10.5846/stxb201102220200
41. Wang J, Zhu L, Daut G, Ju J, Lin X, et al. (2009) Investigation of bathymetry and water quality of Lake Nam Co, the largest lake on the central Tibetan Plateau, China. Limnology 10: 149-158. https://doi.org/10.1007/s10201-009-0266-8
42. Cole TM, Buchak EM (2003) CE-QUAL-W2: A Two-Dimensional, Laterally Averaged, Hydrodynamic and Water Quality Model, Version 2.0. User Manual: Civil and Environmental Engineering Faculty Publications and Presentations.
43. Xie Q, Liu Z, Fang X, Chen Y, Li C, et al. (2017) Understanding the temperature variations and thermal structure of a subtropical deep river-run reservoir before and after impoundment. Water 9: 603. https://doi.org/10.3390/w9080603
44. Dake JM, Harleman DR (1969) Thermal Stratification in Lakes: Analytical and Laboratory Studies. Water Resour. Res.: 484-495. https://doi.org/10.1029/WR005i002p00484
45. Li N, Tuo Y, Deng Y, An R, Li J, et al. (2018) Modeling of thermodynamics of ice and water in seasonal ice-covered reservoir. Journal of Hydrodynamics 30: 267-275. https://doi.org/10.1007/s42241-018-0035-4
46. Li Y, Acharya K, Yu Z (2011) Modeling impacts of Yangtze River water transfer on water ages in Lake Taihu, China. Ecological Engineering 37: 325-334. https://doi.org/10.1016/j.ecoleng.2010.11.024
47. Li Y, Acharya K, Chen D, Stone M (2010) Modeling water ages and thermal structure of Lake Mead under changing water levels. Lake and Reservoir Management 26: 258-272. https://doi.org/10.1080/07438141.2010.541326
48. Oveisy A, Boegman L, Imberger J (2012) Three - dimensional simulation of lake and ice dynamics during winter. Limnology and Oceanography 57: 43-57. https://doi.org/10.4319/lo.2012.57.1.0043
49. Tuo Y, Deng Y, Li J, Li N, Li K, et al. (2018) Effects of dam reconstruction on thermal-ice regime of Fengman Reservoir. Cold Regions Science and Technology 146: 223-235. https://doi.org/10.1016/j.coldregions.2017.08.014
50. Han B, Armengol J, Garcia JC, Comerma M, Roura M, et al. (2000) The thermal structure of Sau Reservoir (NE: Spain): a simulation approach. Ecological Modelling 125: 109-122. https://doi.org/10.1016/s0304-3800(99)00176-3
51. Arfi R (2005) Seasonal ecological changes and water level variations in the Sélingué Reservoir (Mali, West Africa). Physics and Chemistry of the Earth, Parts A/B/C 30: 432-441. https://doi.org/10.1016/j.pce.2005.06.010
52. Stainsby EA, Winter JG, Jarjanazi H, Paterson AM, Evans DO, et al. (2011) Changes in the thermal stability of Lake Simcoe from 1980 to 2008. Journal of Great Lakes Research 37: 55-62. https://doi.org/10.1016/j.jglr.2011.04.001
53. Rueda-Valdivia FJ, Schladow G (2009) Mixing and stratification in lakes of varying horizontal length scales: Scaling arguments and energy partitioning. Limnology & Oceanography 54: 2003-2017.

https://doi.org/10.4319/lo.2009.54.6.2003

54. Ma W, Huang T, Li X, Zhang H, Ju T (2015) Impact of short-term climate variation and hydrology change on thermal structure and water quality of a canyon-shaped, stratified reservoir. Environmental Science and Pollution Research 22: 18372-18380. https://doi.org/10.1007/s11356-015-4764-4

55. Livingstone DM (2003) Impact of secular climate change on the thermal structure of a large temperate central European lake. Climatic change 57: 205-225. https://doi.org/10.1023/A:1022119503144

List of Tables

Table 1. Monitoring scheme

Project	Point Location	Latitude and Longitude	Instrument	Period and Frequency
Continuous water temperature	A1: Wululong<br>A2: Rezhenzangbu	91°12'19"E30°15'51"N<br>91°31'17"E30°18'20"N	ZDR-20h	2016.08.25~2017.09.28, Once per hour.
Meteorological	A3: Dam top	91°21'49"E30°10'55"N	Jinzhou SunshinePC-4	
Vertical water temperature	B1: In front of dam	91°21'09"E30°11'15"N	TidbiT v2（UTBI-001）	2016.08.25~2016.08.31, Once per hour.
Manual monitoring	B1: In front of dam	91°21'09"E30°11'15"N	EXO2	2016.09~2016.12, Once a month

Table 2. Water temperature variation

Date	Air temperature variation /°C	Inflow temperature variation/°C	Water temperature variation/°C								
			0	1	2	3	4	5	10	20	30
2016/8/25	12.3	4.8	5.7	2.6	0.9	0.9	0.8	0.8	0.7	0.5	0.3
2016/8/26	9.6	4.5	3.9	1.5	1.2	1.0	0.6	0.5	1.1	0.3	0.3
2016/8/27	13.4	5.5	5.5	1.4	0.9	0.7	0.5	0.4	0.6	0.5	0.4
2016/8/28	12.6	4.9	5.7	3.0	2.5	1.3	0.8	0.5	0.4	0.4	0.4
2016/8/29	10.3	5.1	3.4	1.4	1.3	1.3	1.0	0.9	1.1	0.4	0.4
2016/8/30	6.8	5.2	2.3	0.9	0.9	1.1	1.2	1.2	0.5	0.4	0.3
2016/8/31	10.5	4.7	2.6	1.1	1.0	1.2	1.3	0.8	0.5	0.3	0.3
Average	10.8	5.0	4.1	1.7	1.3	1.1	0.9	0.7	0.7	0.4	0.3

List of Figures

Fig. 1 Study area diagram.

Fig. 2 Temporal distribution of measured air temperature, solar radiation, and incoming water temperature in the study area.

Fig. 3 Measured vertical water temperature changes from August 25, 2016, to August 31, 2016.

Fig. 4 Comparison of calculated and measured water temperature in front of the dam.

Fig. 5 The variation process of the calculated ice thickness in front of the dam.

Fig. 6 Vertical water temperature (A), gradient (B), buoyancy frequency (C), and water age (D) distribution in the calculation period of the Pangduo Reservoir.

Fig. 7 Longitudinal and vertical two-dimensional water temperature and flow field distribution in the Pangduo Reservoir on August 15, 2017.

Fig. 8 Diurnal distribution of net heat flux in front of the dam and inflow temperature (A) and buoyancy frequency in front of the dam (B) on August 15, 2017.

Fig. 9 Longitudinal and vertical two-dimensional water temperature and flow field distribution in the Pangduo Reservoir on February 15, 2017.

Fig. 10 Comparison of daily average heat budget of surface water between the Pangduo Reservoir (A) and the low-altitude reservoir (B) during the calculated period.

Fig. 11 Air temperature and inflow water temperature distribution of the low-altitude reservoir during the calculated period (A). Comparison of vertical water temperature between the low-altitude reservoir and the Pangduo Reservoir in front of the dam during the calculation period (B). Comparison of SI changes between the Pangduo Reservoir and the low-altitude reservoir (C).

Fig. 12 Comparison of diurnal heat budget of surface water between the Pangduo Reservoir (A) and the low-altitude reservoir (B) on August 15, 2017.

Fig. 13 Comparison of the daily variation in the buoyancy frequency of the water column in each layer in front of the dam in the low-altitude reservoir and the Pangduo Reservoir.

Supporting information

S1 Fig. Interpretation of the winter satellite film of the Pangduo Reservoir.

S2 Fig. Designed operating conditions of the Pangduo Hydropower Station.

S3 Fig. Measured and calculated water temperature scatter plot distribution.

Figure 1 Click here to access/download;Figure;Fig1.tif

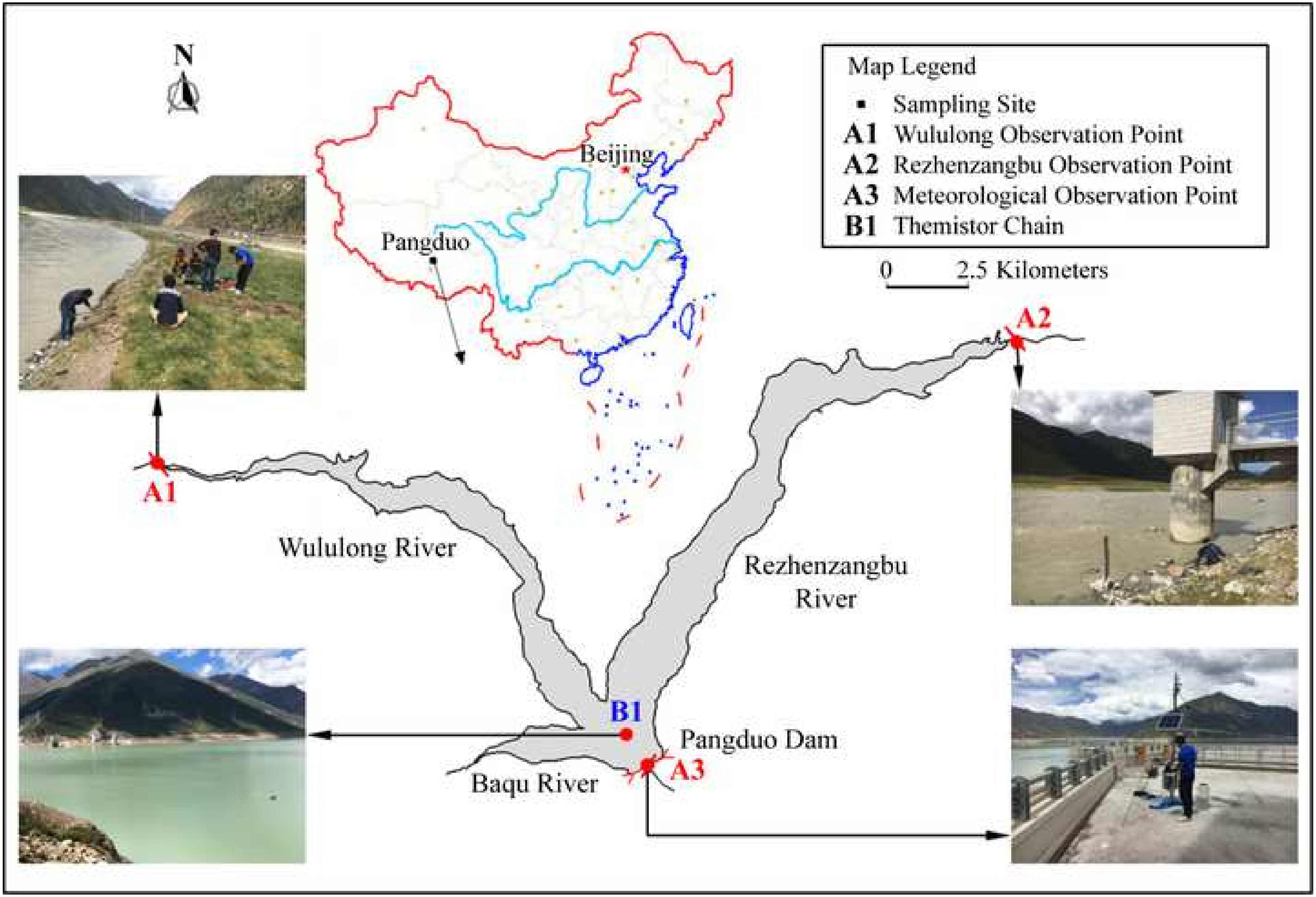

Figure 2

Click here to access/download;Figure;Fig2.tif

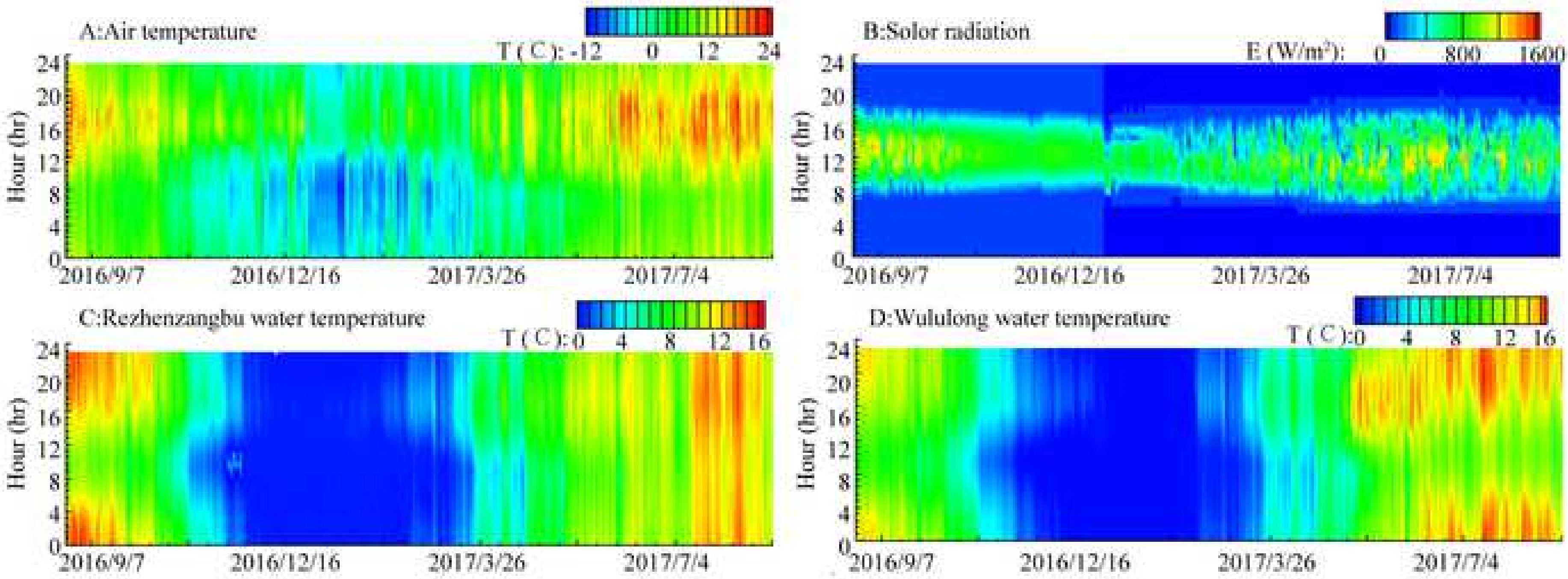

Figure 3

Click here to access/download;Figure;Fig3.tif

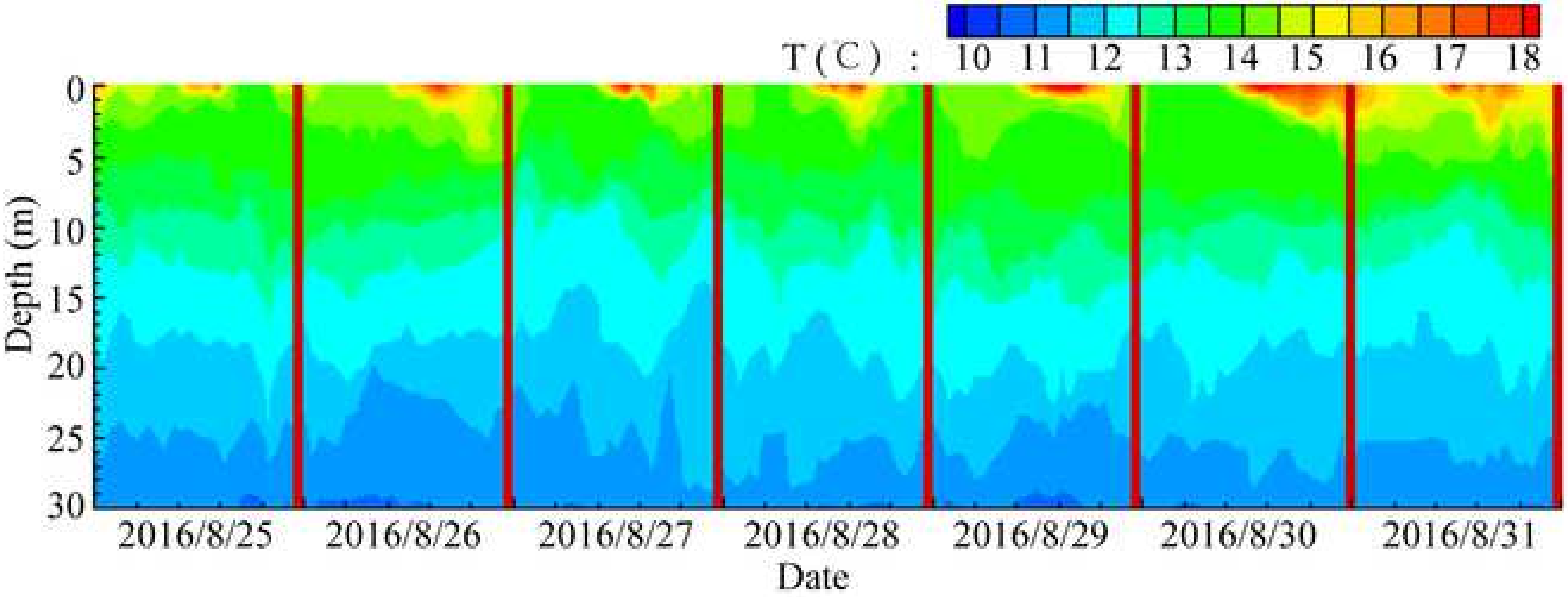

Figure 4

Click here to access/download;Figure;Fig4.tif

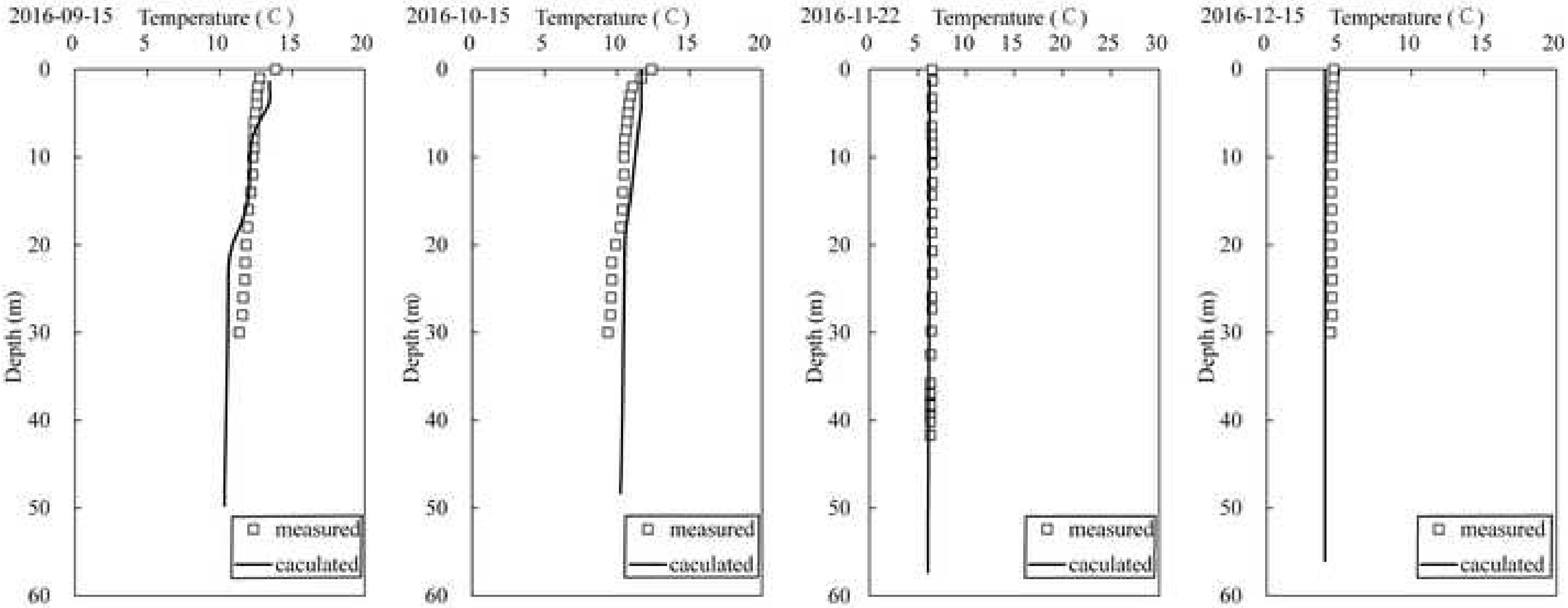

Figure 5

Click here to access/download;Figure;Fig5.tif

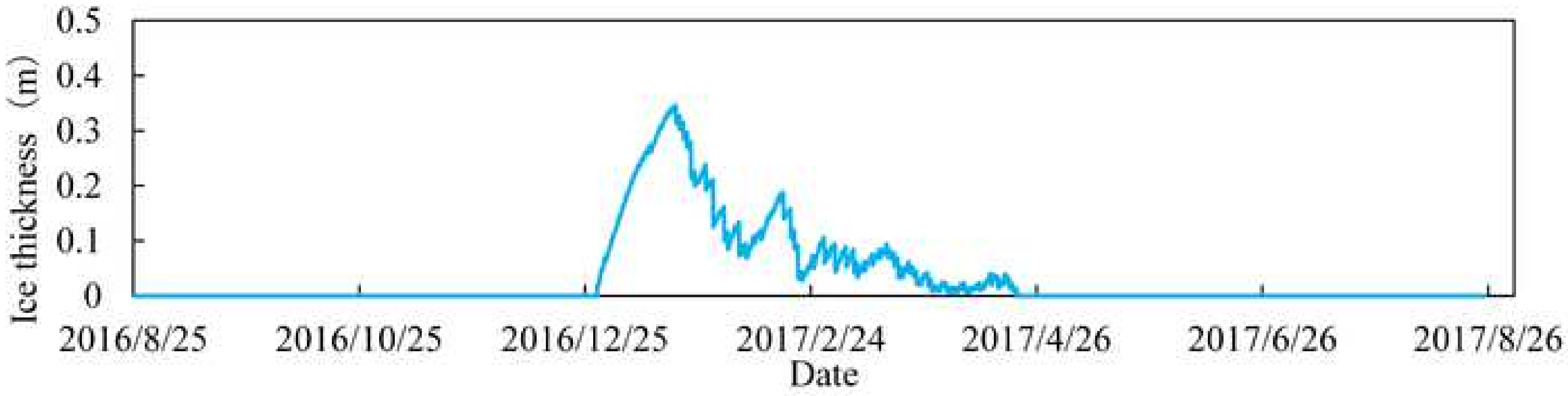

Figure 6

Click here to access/download;Figure;Fig6.tif

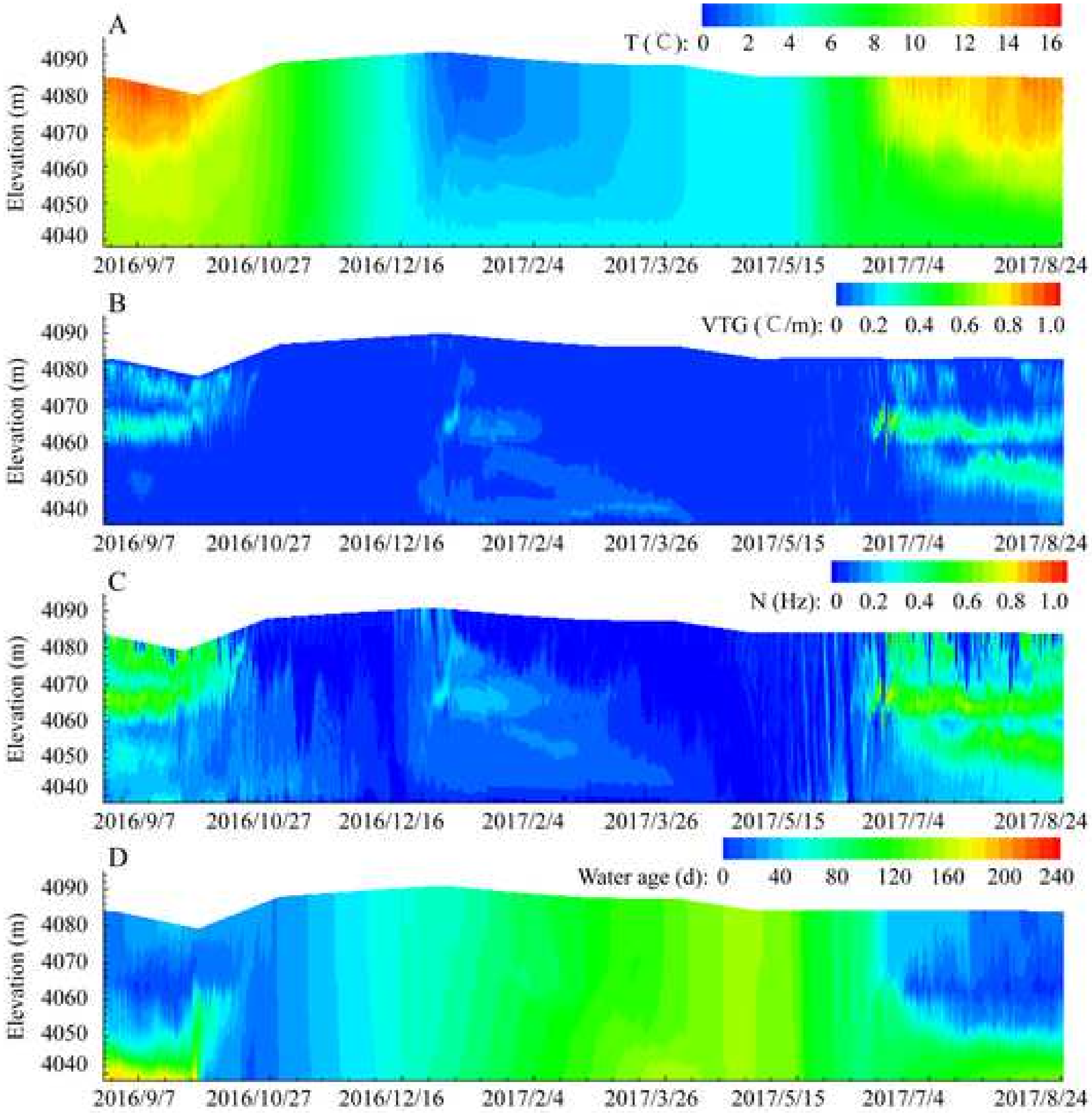

Figure 7

Click here to access/download;Figure;Fig7.tif

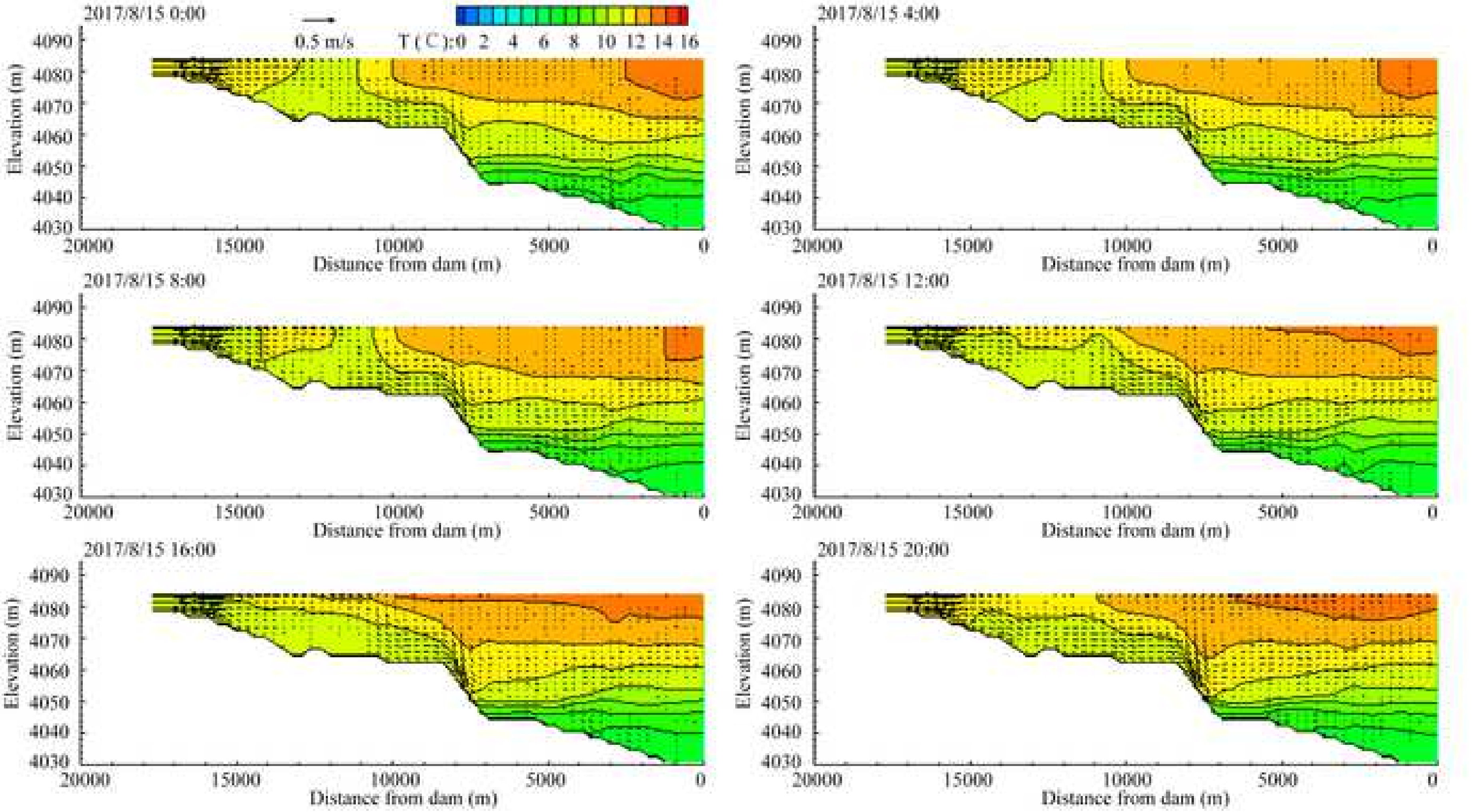

Figure 8

Click here to access/download;Figure;Fig8.tif

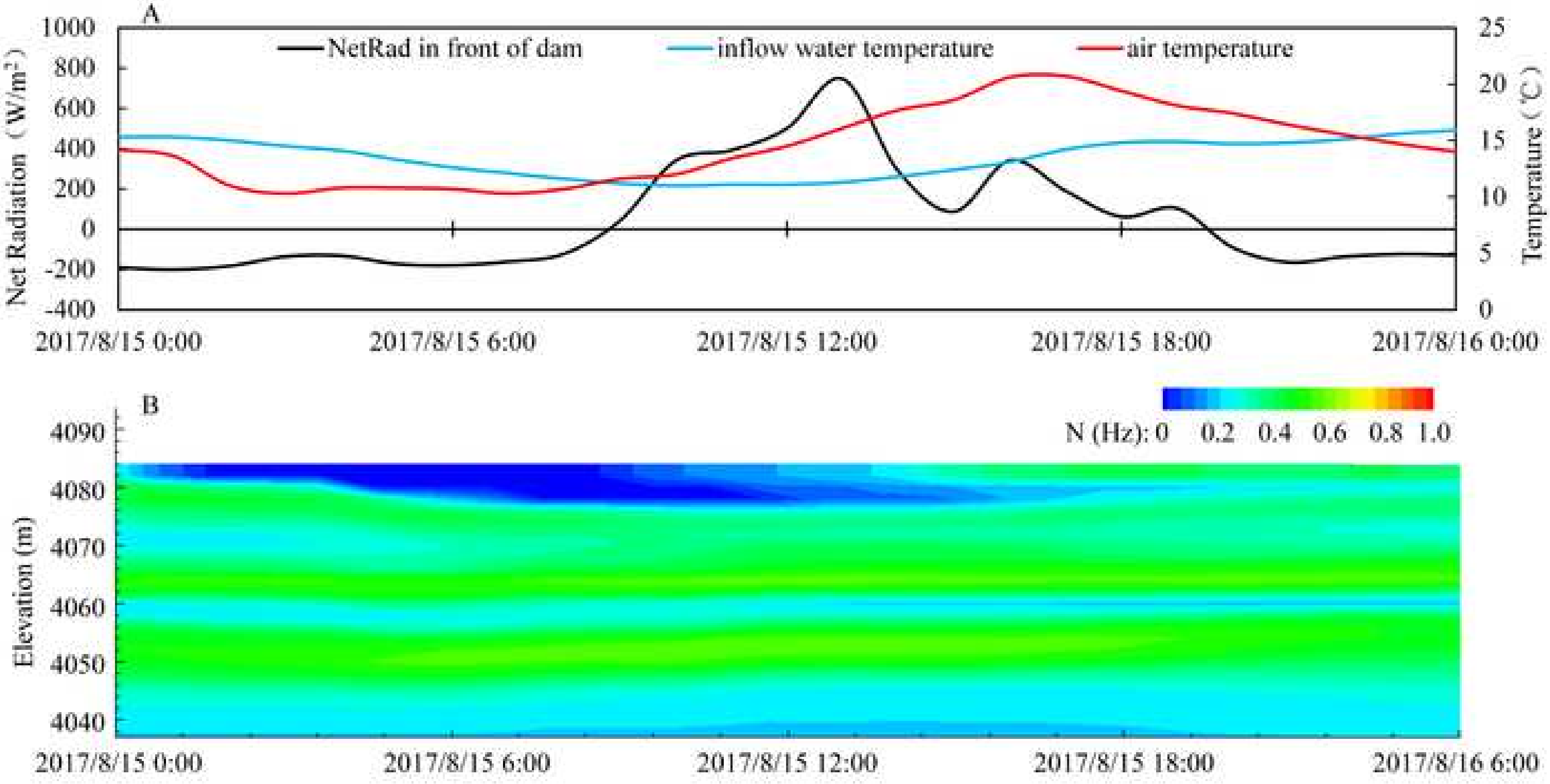

Figure 9

Click here to access/download;Figure;Fig9.tif

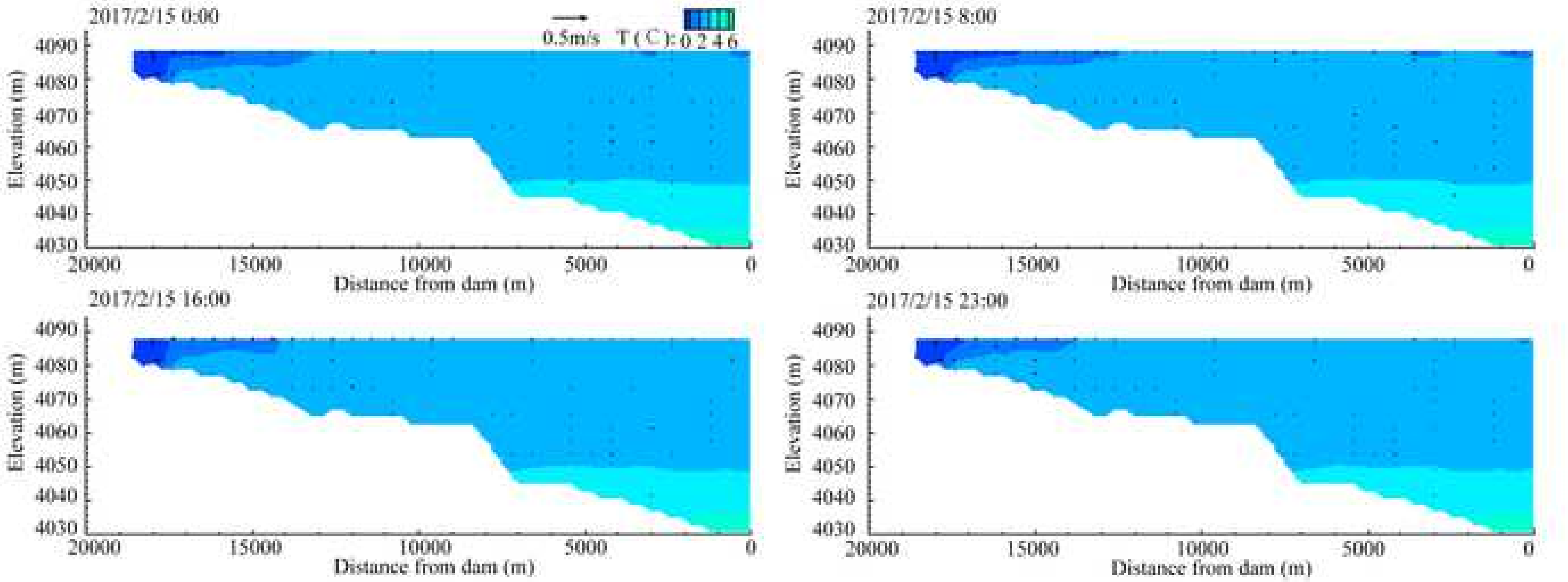

Figure 10

Click here to access/download;Figure;Fig10.tif

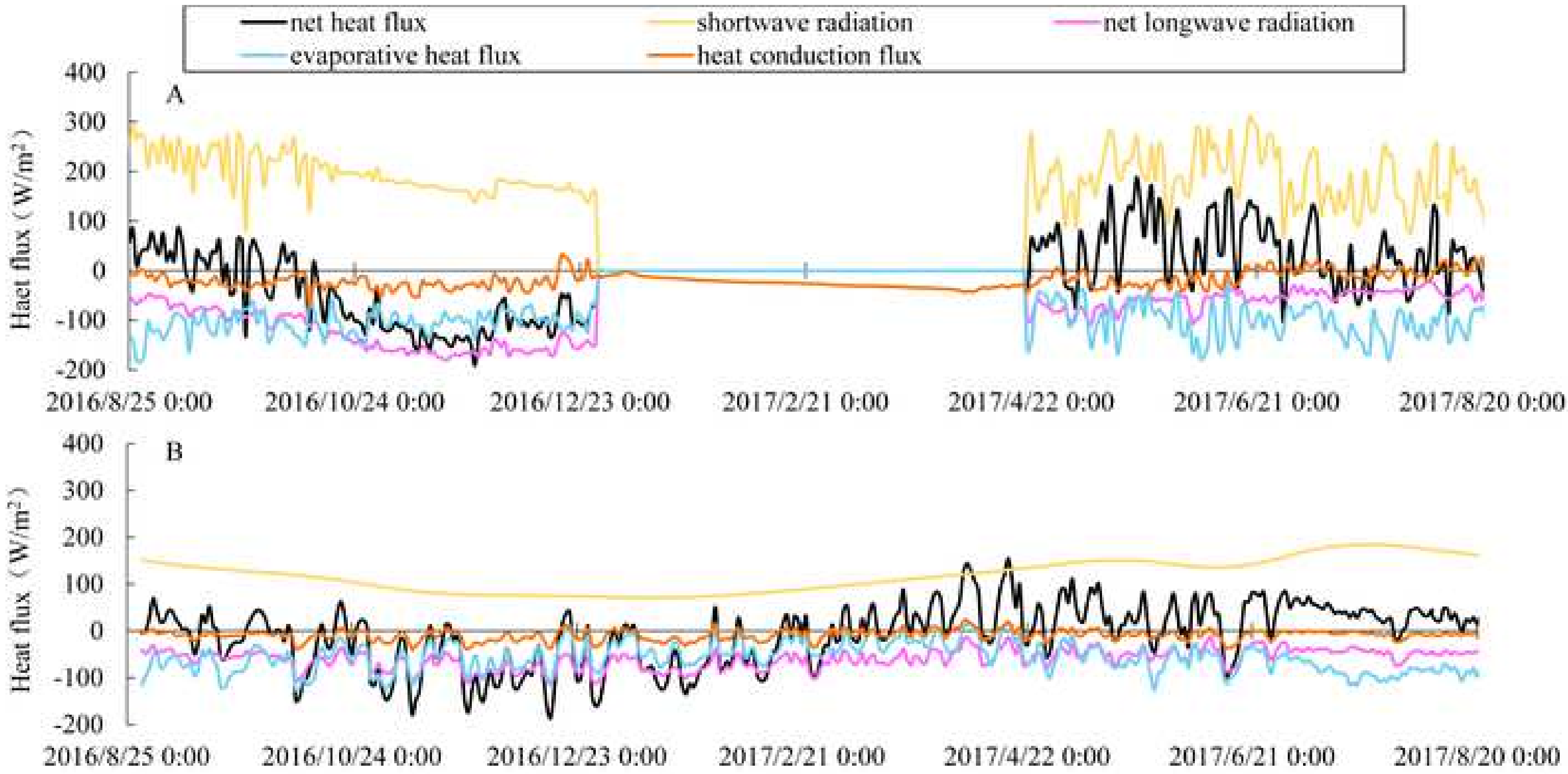

Figure 11

Click here to access/download;Figure;Fig11.tif

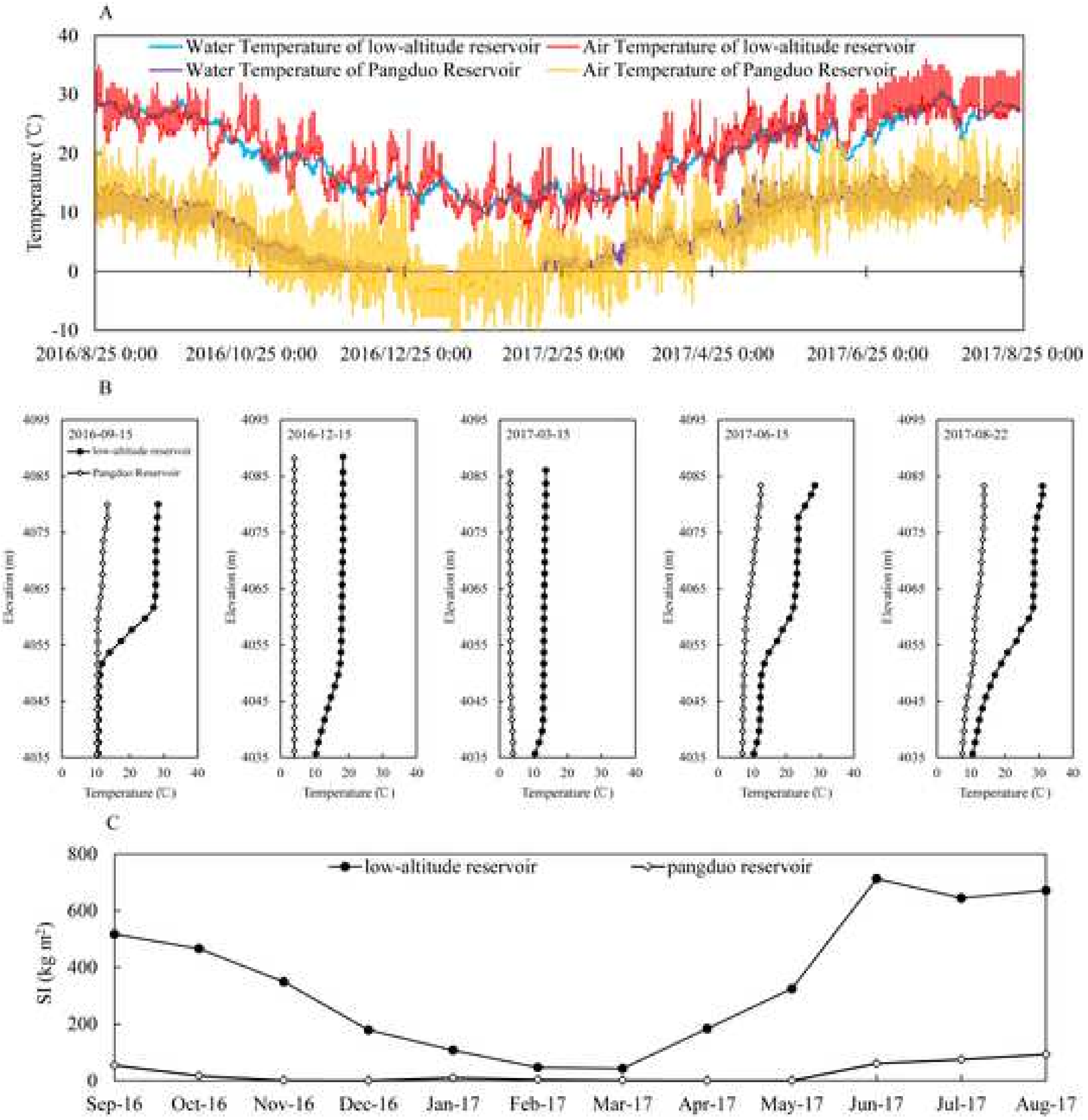

Figure 12

Click here to access/download;Figure;Fig12.tif

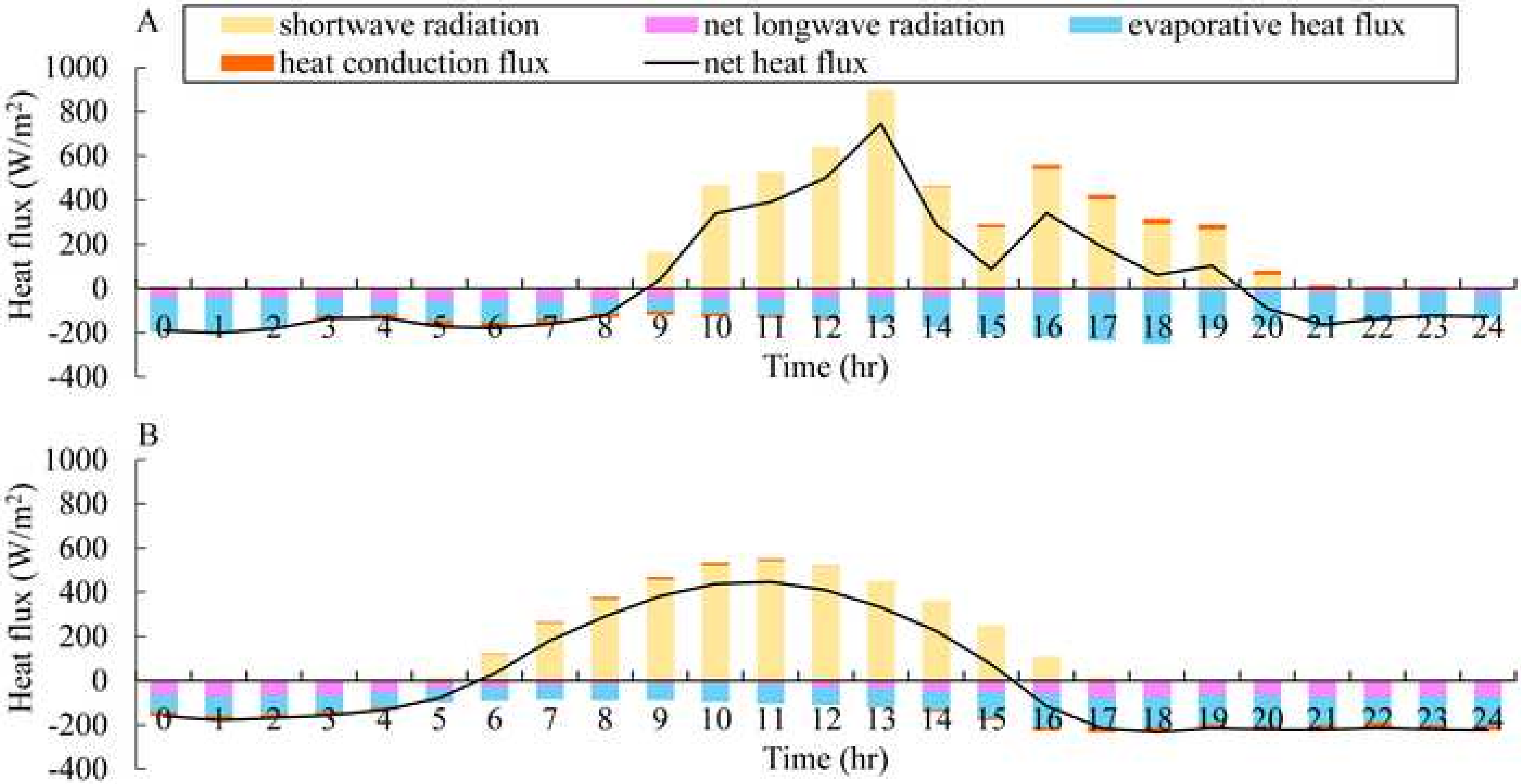

Figure 13

Click here to access/download;Figure;Fig13.tif

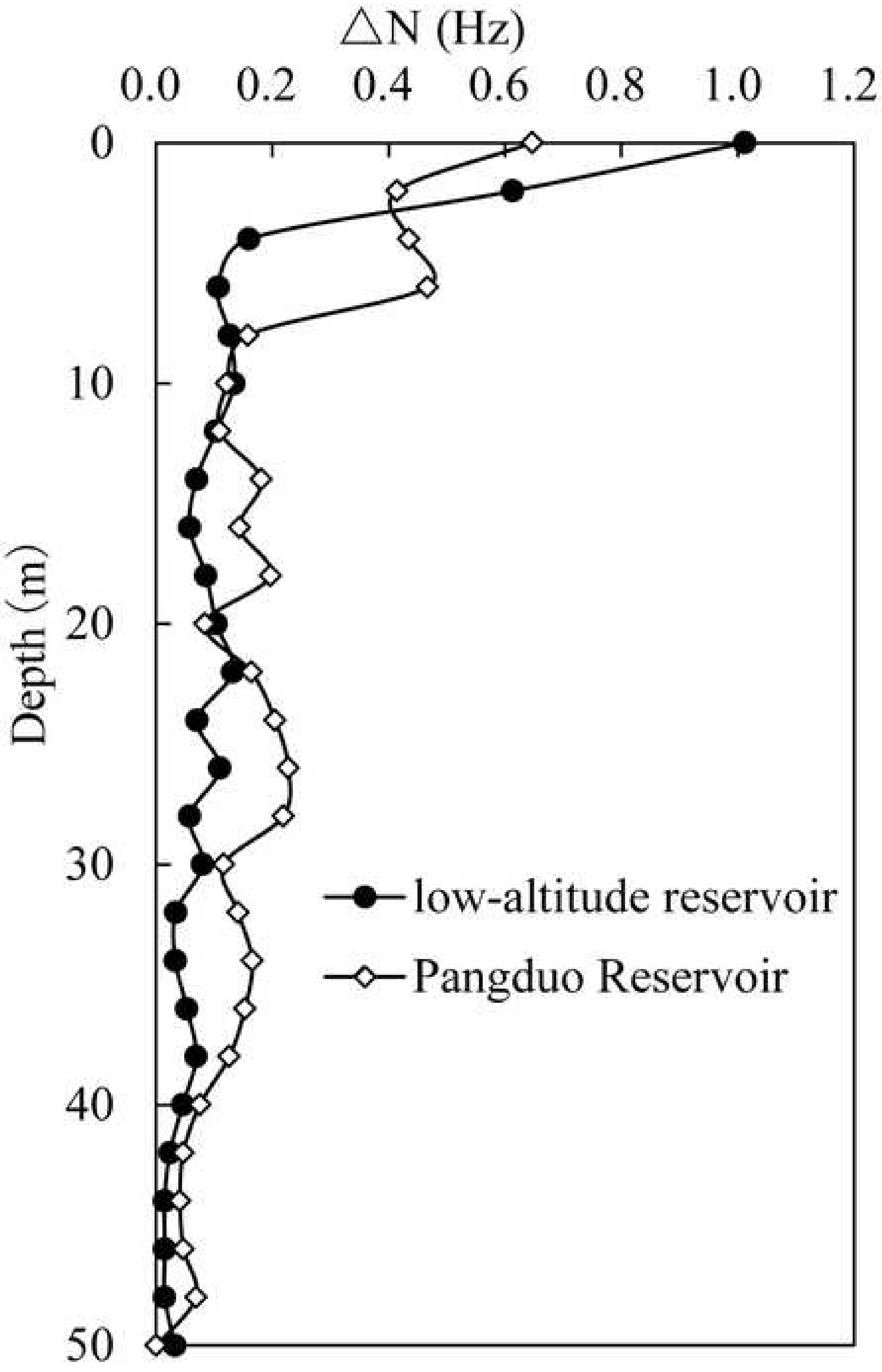

Supporting Information S1 Fig

Click here to access/download
Supporting Information
S1_Fig.tif

Supporting Information S2 Fig

Click here to access/download
Supporting Information
S2_Fig.tif

Supporting Information S3 Fig

Click here to access/download
Supporting Information
S3_Fig.tif
