# Supplementary material for: Study of the thermal regime of a reservoir on the Qinghai-Tibetan Plateau, China
Source: PLoS One. 2020 Dec 21;15(12):e0243198. doi: 10.1371/journal.pone.0243198 (PMC7751983; doi:10.1371/journal.pone.0243198)
Supplement: Attachment — Submitted filename: Review comments.docx [file pone.0243198.s013.docx]

Study of the thermal regime of a reservoir on the Qinghai-Tibet Plateau, China

Review comments:

The CE-QUAL-W2 model is used to explore the typical thermal regime of a stratified reservoir on the Qinghai-Tibetan Plateau (Pangduo Reservoir). The study is interesting, and the modeling results are useful for the scientific understanding of the thermal dynamics in stratified reservoirs under the special geographical and meteorological conditions on the Qinghai-Tibetan Plateau. The paper is well organized, and the results are well presented and discussed. However, after screening the manuscript, I think there are still some points that can be improved. The detailed comments are as follows:

(1) In the Introduction, the CE-QUAL-W2 model should be mentioned in line 14 as it is a famous model.
(2) In lines 72-79, the authors should briefly list the reasons that why CE-QUAL-W2 model is used.
(3) I suggest the authors to summarize all the governing equations (1-8) to a table.
(4) Model grid deserves a figure.
(5) The authors should improve the conclusion section to make it more precise.

Based on the above comments, a minor revision is needed.
