# Supplementary material for: Study of the thermal regime of a reservoir on the Qinghai-Tibetan Plateau, China
Source: PLoS One. 2020 Dec 21;15(12):e0243198. doi: 10.1371/journal.pone.0243198 (PMC7751983; doi:10.1371/journal.pone.0243198)
Supplement: Attachment — Submitted filename: Response to Reviewers3.docx [file pone.0243198.s016.docx]

Dear reviewer,

We greatly appreciate the constructive comments and suggestions. We have reorganized the manuscript as you suggested.

(1) We rewrote the Abstract in a quantitative way according to your comments, defined the objective and highlighted our findings.
(2) We performed a paired t test to analyze the relevant results and reported the *P* values.
(3) We carefully considered your suggestions for the Introduction and modified it with the required sections as you mentioned.
(4) We supplemented the calibration process in the manuscript and the relevant figure and explained the sensitivity of the simulations.
(5) We reorganized the structure of the manuscript and moved some figures (Figs 2-5) to the supplementary material file according to your comments.

Our point-by-point responses to your comments are below.

***1):*** ***Lines 14-16:*** ***Provide numerical results here.***

Response to reviewer’s comment No. 1:

Thank you for this suggestion. We modified it with quantitative results in line 13-14.

***2):*** ***Lines 16-17: What was the dT? What was the percent change compared to winter time?***

Response to reviewer’s comment No. 2:

Thank you for your comment. We added the results of the analysis of variance here for illustration purposes in lines 18-19.

***3): Line 18: What was the number?***

Response to reviewer’s comment No. 3:

We added the results of the analysis of variance in lines 20-22.

***4): Lines 19-20: Provide the percentages.***

Response to reviewer’s comment No. 4:

Thank you for your suggestion. We added percentages and the *P* values of the paired t test in lines 22-25.

***5): Lines 20-21: Again support this argument with quantitative results.***

Response to reviewer’s comment No. 5:

Thank you for your comment. We rewrote this part in lines 25-26.

***6): Line 8: Please see my comments in the 1st page.***

Response to reviewer’s comment No. 6:

Thank you for your constructive recommend. We reorganized it according to your comments. We clarified the research objective and key findings and referenced the results in a quantitative way (lines 9-29).

***7):*** ***Line 28: Checking your references in this paragraph, they are mostly old and out of date. Use some newly published papers in the field, here are some examples:***

***https://doi.org/10.1016/j.jenvman.2019.110023***

***https://doi.org/10.1016/j.scitotenv.2019.03.248***

***https://doi.org/10.3390/w11051060.***

Response to reviewer’s comment No. 7:

Thank you so much for your reminder and recommendations. We added these recommended references and replaced some outdated references in the Introduction (references 2-6, 8, 11, 45, 47-49).

***8):*** ***Line 75: Mention the name of the model in the abstract as well***

Response to reviewer’s comment No. 8:

We mentioned the name in the Abstract in line 11.

***9):*** ***Line 92: You should provide a more informative caption and clearly mention the location of the reservoirs separately in the map and in the caption.***

Response to reviewer’s comment No. 9:

Thank you for your suggestions. We rewrote this part with more specific information, defined the location in lines 102-113, and re-drew Fig 1.

***10):*** ***Line 117: This in the 3rd time I am seeing this in the text. You should avoid redundancy in the text. Consider this and similar issues throughput the manuscript.***

Response to reviewer’s comment No. 10:

Thank you so much for your suggestions. We have reorganized this paragraph in lines 151-158 and checked and revised similar issues in the manuscript carefully. We have also sent our manuscript to a professional English editing service (American Journal Experts (AJE), USA) to improve the English throughout the manuscript.

***11):*** ***Line 120: Provide a proper citation for the model.***

Response to reviewer’s comment No. 11:

We have added the reference (Reference 46) and revised the text in line 153.

***12):*** ***Line 126: If you haven't updated any of the following equations, you don't have to provide all of them as they are already published and accessible. You can remove some or all of them or send them to a supplementary material file to save some space.***

Response to reviewer’s comment No. 12:

Thank you so much for your suggestions. The governing equations were still retained for easy of reference, but we have summarized the governing equations (1-8) to Table 2 to save space.

***13): Line 150: How about calibration?***

Response to reviewer’s comment No. 13:

We have supplemented the calibration process in lines 210-217 in the manuscript and the relevant figure (S6 Fig), and explained the sensitivity coefficient of the simulations.

***14):*** ***Line 161: Provide citation for this and Eq. 10.***

Response to reviewer’s comment No. 14:

Thank you for your reminder. We have added it in lines 175-176 with reference 50 and 51.

***15):*** ***Line 182: Needs a space. Check all the document for similar issues.***

Response to reviewer’s comment No. 15:

Thank you for your reminder. We have added it in line 129 and checked and revised similar issues in the manuscript.

***16):*** ***Line 183: Add degree C.***

Response to reviewer’s comment No. 16:

We have added it in line 129 and checked and revised similar issues in the manuscript.

***17):*** ***Lines 182-194: This is not part of your results. It should be presented in the Methods section and the figures also could be presented in the supplementary materials file.***

Response to reviewer’s comment No. 17:

Thank you for your reminder. We have moved this information to the section ***Regional monitoring*** in lines 129-138. The figures are now presented in the Supporting information (S1 Fig).

***18):*** ***Line 196: Explain the panels in the caption.***

Response to reviewer’s comment No. 18:

Thank you for your reminder. We have explained the panels in the captions in line 596.

***19):*** ***Lines 216-217: Provide the numerical results here as well.***

Response to reviewer’s comment No. 19:

Thank you for your reminder. We have provided the numerical results in lines 225-226.

***20):*** ***Lines 210-211: Figure 4 only includes 4 panels without further information on why these 4 days are selected in the period. How was the performance of the model in calibration compared to the validation? If these values in fig. 4 are the average daily or observed for a time. Overall this figure is confusing and should be deleted. You need to think of a better way of visualizing this.***

Response to reviewer’s comment No. 20:

Thank you for your comments. We have explained why these 4 days are selected in lines 218-220. The validation period included the high temperature period in summer and the low temperature period before freezing. The calibrated data were measured by EXO2 (Table 1) during the manual monitoring time, and we explained the data source in lines 218-219. In the model verification, we added the vertical water temperature comparison between August 25 and August 30. In addition, we redrew Figure 4 to clarify the visual presentation (S7 Fig) and re-analyzed the errors in lines 222-224.

***21): Lines 222-229: This is a part of the methods for me.***

Response to reviewer’s comment No. 21:

Thank you for your comments. This part was actually a verification of the ice period. We did not measure data during the winter icing period because of engineering limitations and researcher safety considerations. Therefore, we obtained the ice process in the study area through satellite pictures, and ice was also considered in the model. The time of freezing and melting was consistent, which showed that the process of thermal budget was consistent. We rewrote this part in lines 229-232 and moved Figure 4 to the supporting information (S9 Fig).

***22):*** ***Lines 233-234: Present this in a quantitative way.***

Response to reviewer’s comment No. 22:

Thank you for your comments. We rewrote this sentence in lines 236-239 using quantitative results.

***23):*** ***Lines 279-280: By what percent?***

Response to reviewer’s comment No. 23:

Thank you for your comments. We rewrote this information based on the variance analysis results in lines 281-282 and reorganized this paragraph.

***24):*** ***Line 327: Provide relevant information for Fig. 10 first.***

Response to reviewer’s comment No. 24:

We carefully checked and revised the order of the pictures to ensure that the order of the pictures and text matches.

***25):*** ***Line 331: The panels are too small and again, there is not explanation why and how these time windows are selected.***

Response to reviewer’s comment No. 25:

Thank you so much for your suggestion. We redrew Figure 4 and enlarged the panel according to your comments. We explained why and how these time windows are selected in lines 327-328 and used the recommended paired t-test to analyze this part of the data and reported the *P* value, and the results were reorganized and discussed in lines 343-351.
