# Supplementary material for: Study of the thermal regime of a reservoir on the Qinghai-Tibetan Plateau, China
Source: PLoS One. 2020 Dec 21;15(12):e0243198. doi: 10.1371/journal.pone.0243198 (PMC7751983; doi:10.1371/journal.pone.0243198)
Supplement: Attachment — Submitted filename: Response to Reviewers.docx [file pone.0243198.s017.docx]

Dear reviewers,

We are grateful to the editor for giving us the opportunity to further improve manuscript PONE-D-20-19898R1, entitled “Study of the thermal regime of a reservoir on the Qinghai-Tibetan Plateau, China”. We are grateful for the constructive comments and suggestions, which were carefully considered in this minor revision of our manuscript.

Our point-by-point responses to your comments are provided below.

***1):*** ***Line 14: please specify what frequency of the observed data is used to calculate RMSE? Hourly, daily, or monthly?***

Response to reviewer comment No. 1:

Thank you for this comment. We specified the frequency of the observed data in lines 13-14.

***2):*** ***Line 90: What do you mean by the “river-run reservoir”? Do you mean “run-of-river reservoir”? Based on the data you showed in Figure 2d, I would argue whether this reservoir is a run-of-the-river reservoir. It is more like a storage reservoir. Commonly, run-of-the-river reservoirs are barely stratified.***

Response to reviewer comment No. 2:

Thank you for your constructive comment. We meant “run-of-river reservoir”, and this was modified in line 91. Most of the inflow of run-of-river reservoirs enters from the main river and forms a plug flow with a mainstream direction. These reservoirs have a larger flow, a smaller volume and a mainstream layer, and the retention time is smaller than that of storage reservoirs and lakes. The Pangduo Reservoir has these characteristics. With the expansion of reservoir construction, these large run-of-river deep reservoirs will also have temperature stratification, such as that in the Xiluodu Reservoir (Fig 1 below), and the water age of the hypolimnion varies between 100 and 300 days (Fig 2 below). The specific references are listed below.

Fig 1. Simulated water temperature at a cross-section 3.5 km upstream to the Xiluodu Dam in reference 1.

Fig 2. Simulated water age at a cross-section 3.5 km upstream to the Xiluodu Dam in reference 1.

References:

Reference 1. Xie, Q., Liu, Z., Fang, X., Chen, Y., Li, C., MacIntyre, S., 2017. Understanding the Temperature Variations and Thermal Structure of a Subtropical Deep River-Run Reservoir before and after Impoundment. Water-Sui. 9(8), 603.

Reference 2. Hayes, N.M., Deemer, B.R., Corman, J.R., Razavi, N.R., Strock, K.E., 2017. Key differences between lakes and reservoirs modify climate signals: A case for a new conceptual model. Limnology & Oceanography Letters 2(2).

Reference 3. Naderi, V., Farsadizadeh, D., Dalir, A.H., Arvanaghi, H., 2014. Effect of Using Vertical Plates on Vertical Intake on Discharge Coefficient. Arabian Journal for Science & Engineering 39(12), 8627-8633.

Reference 4. Akiyama, J., Stefan, H.G., 1987. Gravity Currents in Lakes, Reservoirs and Coastal Regions: Two-Layer Stratified Flow Analysis. St Anthony Falls Laboratory.

***3): Line 111: “…reservoir is 12.3 x 108 m3 [at] (under) the 4095 m a.s.l.”***

Response to reviewer comment No. 3:

Thank you for your reminder. This was modified in line112 according to your comment.

***4): Equation 11 is not necessary. The authors can simply state that in text.***

Response to reviewer comment No. 4:

Thank you very much for your suggestion. We deleted Equation 11 and stated the content in the text in lines 199-202.

***5):*** ***Section 3.4: I would recommend the authors have a summary paragraph to summarize what statistical experiments they did in this study. The current paragraph is oversimplified, and I am not particularly interested in what software the authors used to do this analysis; Python, R, Excel does not make any difference.***

Response to reviewer comment No. 5:

Thank you for your constructive comment. We revised the paragraph to summarize the statistical experiments performed in the study in lines 204-209.

***6): Line 223: Add units to standard deviation. Additionally, it is unnecessary to report a relative error in river temperatures. Unlike precipitation, zero precipitation means there is no precipitation, but zero degrees Celsius still has physical meanings.***

Response to reviewer comment No. 6:

Thank you for your reminder. We added units to the standard deviation in line 234 and performed similar edits throughout the manuscript. We agree with you that reporting the relative error is unnecessary, and this was deleted.

***7):*** ***Please rearrange the supplemental figures as they appear in the main text.***

Response to reviewer’s comment No. 7:

Thank you for your reminder. We rearranged and carefully checked the order of supplementary figures in the manuscript.

***8):*** ***8. Line 236: I would recommend using the term “turnover” rather than “inversion”.***

Response to reviewer comment No. 8:

We modified this term in line 246, line 248 and line 430.

***9):*** ***Line 280: “…fluctuated [between] (from) XX °C [and] (to) XX°C.” The temperature fluctuation is not monotonic.***

Response to reviewer comment No. 9:

Thank you very much for your reminder. This was modified in line 290 and line 309.

***10):*** ***The first paragraph in Section 4.5 belongs to methodology.***

Response to reviewer comment No. 10:

Thank you for your comment. We moved the first paragraph in section 4.5 to the methodology section, specifically, the last paragraph of section 3.2, lines 174-178.

***11):*** ***It would recommend that the authors further discuss how this study's findings can have a broader impact. It would make this study more impactful.***

Response to reviewer comment No. 11:

Thank you for your constructive comment. We added section 4.6 and related references to discuss the impact and scientific significance of this research and discussed future work related to this study specifically in lines 409-427.
